# The therapeutic potential of Zuogui Wan in oligoasthenozoospermia: insights from network pharmacology, molecular docking, molecular dynamics simulation, and experimental validation

**DOI:** 10.1038/s41598-025-22348-w

**Published:** 2025-11-04

**Authors:** Mingzhao Zhang, Jisheng Wang, Junlong Feng, Baojun Ju, Jingxun Yang, Zhenfei Gao, Xiangyu Wang, Shuxi Zhou, Xiao Li

**Affiliations:** 1 Urology, Henan Second Provincial People’s Hospital, Zhengzhou, Henan China; 2 Department of Andrology, The First Affiliated Hospital, Henan University of Chinese Medicine, Zhengzhou, 450000 Henan China; 3 First School of Clinical Medicine, The First Affiliated Hospital, Henan University of Chinese Medicine, Zhengzhou, Henan China; 4 Department of Andrology, Dongzhimen Hospital, Beijing University of Chinese Medicine, Beijing, China; 5 Beijing Changping District Hospital of Traditional Chinese Medicine, Beijing, 102200 China

**Keywords:** Zuogui wan, Oligoasthenozoospermia, Network pharmacology, Molecular docking, Molecular dynamics simulation, PI3K-AKT pathway, MAPK pathway, Cell apoptosis, Diseases, Molecular medicine, Urology

## Abstract

Oligoasthenozoospermia (OAS) is a major cause of male infertility, with limited effective treatments. Chinese patent medicine Zuogui Wan (ZGW) has been traditionally used to improve sperm quality, but its molecular mechanisms remain unclear. This study integrates network pharmacology, molecular docking, molecular dynamics (MD) simulation, and in *vivo* and in *vitro* experiments to explore ZGW’s therapeutic effects in OAS. Active compounds and targets of ZGW were identified using network pharmacology, and intersecting OAS-related targets underwent enrichment and protein-protein interaction (PPI) analysis. Molecular docking and MD simulations assessed compound-target binding affinity and stability. In *vitro*, CCK-8 assays measured cell proliferation, while qPCR and Western blot analyzed key gene and protein expression. In *vivo*, a rat OAS model was used to evaluate ZGW’s therapeutic effects through transmission electron microscopy (TEM), hematoxylin & eosin (HE) staining, and TUNEL assays. The expression of key molecular targets was further validated by qPCR and Western blot. A total of 182 potential targets were identified, with TP53, NF-κB1, and PKC as key hub genes. KEGG pathway analysis highlighted the involvement of the PI3K-AKT and MAPK signaling pathways.Four core bioactive compounds—Cyasterone, Betavulgarin, Kaempferol, and Quercetin—were identified, with Cyasterone exhibiting the strongest binding affinity and highest stability.In *vitro* experiments demonstrated that ZGW significantly promoted cell proliferation and regulated apoptosis-related gene expression, indicating its potential in enhancing sperm function. In *vivo*, ZGW improved testicular structure, enhanced sperm quality, and reduced spermatogenic cell apoptosis, as evidenced by TEM, HE, and TUNEL assays. Molecular validation further confirmed ZGW’s modulation of key signaling pathways involved in OAS. ZGW modulates apoptosis, oxidative stress, and key pathways (PI3K-AKT, MAPK) while regulating TP53, NF-κB, and PKC expression. Cyasterone exhibits strong binding and stability with core targets. This study supports ZGW as a potential treatment for male infertility.

**Supplementary Information:**

The online version contains supplementary material available at 10.1038/s41598-025-22348-w.

## Introduction

Over the past five decades, there has been a marked decline in sperm count and quality across multiple regions, including North America, Europe, Australia, and New Zealand^1^. This decline has contributed to a steady rise in cases of oligoasthenozoospermia (OAS), a leading cause of male infertility characterized by reduced sperm concentration and motility^2^. Spermatogenic dysfunction is a key factor in male infertility and is closely associated with a variety of intrinsic and extrinsic factors, such as varicocele, reproductive tract infections, endocrine disorders, and environmental pollutants^3^. Despite advancements in modern medicine, the diagnosis and treatment of male infertility remain challenging^4^, with approximately 60% to 75% of men with OAS unable to receive a definitive diagnosis after thorough evaluation^5^. Current clinical interventions primarily focus on symptom management, with common treatments including L-carnitine, kallikrein, coenzyme Q10, lipoic acid, vitamin E, and varicocelectomy^6^. However, these therapies often yield inconsistent clinical outcomes, and some lack approval in international guidelines^7^. Furthermore, while assisted reproductive technologies have shown effectiveness, they are associated with potential adverse effects, such as drug dependence and reproductive system damage, limiting their broader application^8,9^.

Therefore, understanding the mechanisms underlying the establishment and maintenance of male fertility and developing effective treatments for spermatogenic dysfunction have become urgent priorities. Traditional Chinese Medicine (TCM) is widely recognized for its simple, effective, cost-efficient, and holistic therapeutic approaches, and its role in treating male infertility has gained increasing international recognition^10^. In particular, kidney-tonifying therapies in TCM have demonstrated clinical efficacy and are widely accepted by patients worldwide. Zuogui Wan(ZGW), a classic kidney-tonifying and sperm-promoting herbal formula, has shown remarkable therapeutic effects in treating male infertility, particularly oligoasthenozoospermia. Originating, this formula nourishes kidney yin, replenishes essence, and benefits the marrow. It has been widely used as a proprietary Chinese medicine in China, Japan, and other countries^11^. Modern medical research has revealed that ZGW significantly improves sperm quality in OAS patients, regulates reproductive hormone levels, enhances testicular tissue morphology, and promotes spermatogonial proliferation^12^. Additionally, it has been shown to improve spermatogenesis in animal models^13^ and inhibit apoptosis^14^, underscoring its therapeutic potential for male infertility.

**Fig. 1:**
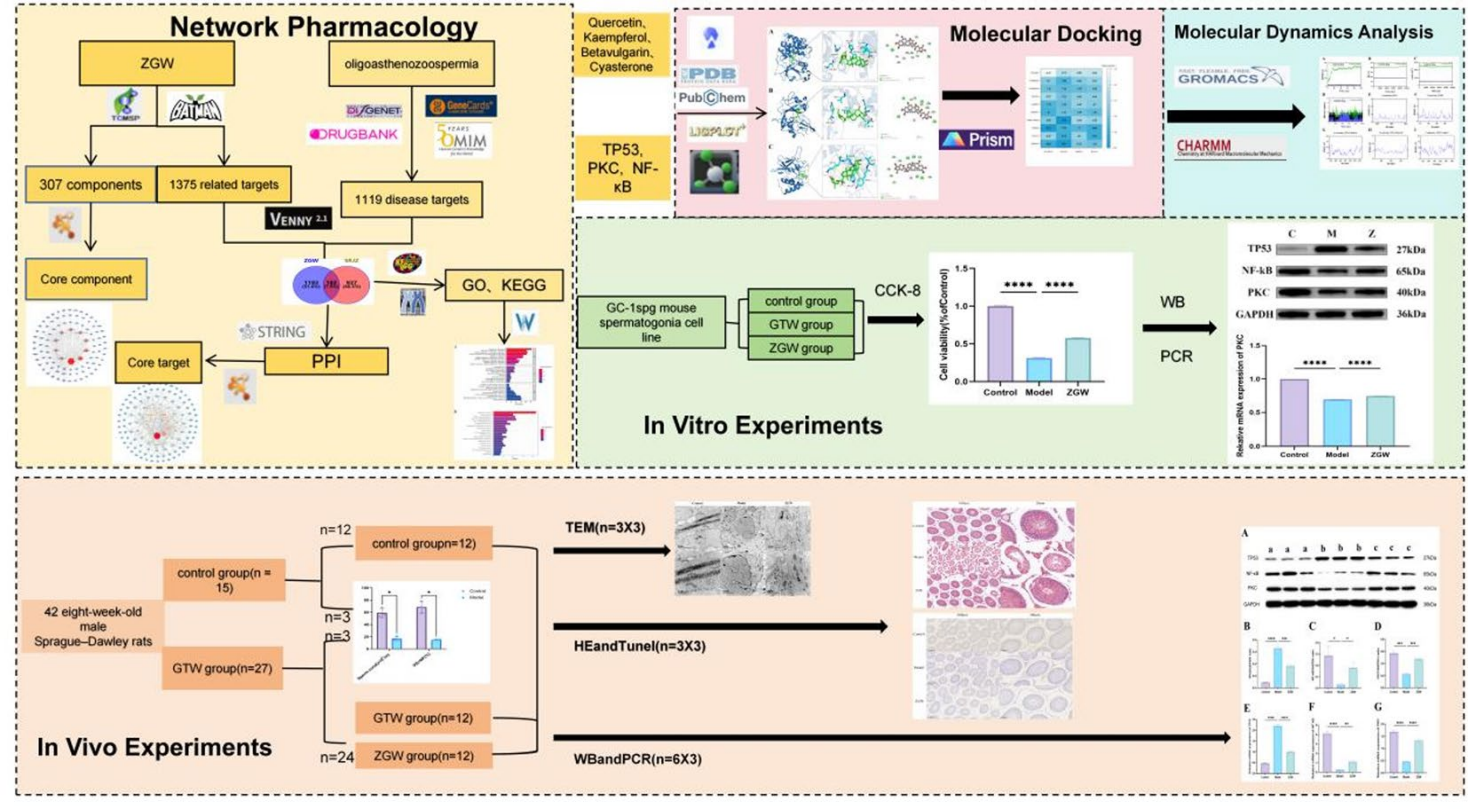
The potential treatment flowchart of Zuo Gui Wan for oligoasthenozoospermia.

The network pharmacology model proposed by Li Shao has revolutionized the study of drug–disease interactions by integrating biological networks to identify drug targets and elucidate mechanisms of action^15^. This approach represents a paradigm shift in TCM research, moving from a “single-drug, single-target” perspective to a systems-level analysis^16^. Network pharmacology not only enables a comprehensive exploration of the intricate interactions between drugs, genes, and targets—enhancing our understanding of TCM’s mechanisms of action—but also strengthens its clinical applicability^17^. By constructing detailed network topologies, this method clarifies the compatibility mechanisms of herbal formulas, providing a solid scientific foundation for optimizing classical prescriptions^18^.This study focuses on ZGW, investigating its multi-component, multi-target, and multi-pathway mechanisms at the molecular level. Through network pharmacology analysis, molecular docking, and in vivo and in vitro experimental validation, we elucidate the potential mechanisms underlying ZGW’s therapeutic effects on oligoasthenozoospermia, thereby deepening our understanding of its efficacy and clinical application potential.The flowchart of the entire experiment is shown in Fig. 1.

## Materials and methods

### Network pharmacology

#### Screening of active compounds and targets of ZGW

Since no data on Tortoise Plastron Glue (Gui Ban Jiao) and Deer Antler Glue (Lu Jiao Jiao) were available in the Traditional Chinese Medicine Systems Pharmacology database and analysis platform(TCMSP) database17 (https://www.tcmsp-e.com/), relevant compounds were retrieved using the keywords “CHUAN NIU XI,” “GOU QI ZI,” “SHAN YAO,” “SHAN ZHU YU,” “TU SI ZI,” and “SHU DI HUANG.” Screening criteria were set as drug-likeness score ≥ 0.18 and oral bioavailability ≥ 30% to identify the chemical constituents of ZGW.

To identify bioactive compounds of Zuogui Wan, we used two pharmacokinetic parameters: oral bioavailability (OB) and drug-likeness (DL), both obtained from the Traditional Chinese Medicine Systems Pharmacology Database (TCMSP). OB was predicted using the OBioavail 1.1 model, which integrates metabolic (CYP3A4) and transport (P-glycoprotein) properties to simulate the absorption, distribution, and disposition of compounds^19^. Compounds with OB ≥ 30% were selected as they are considered to possess acceptable oral absorption and systemic exposure^20^.

DL was calculated by the Tanimoto similarity between the molecular descriptors of each compound and the average molecular descriptors of all approved drugs in the DrugBank database^17^. The mean DL value of approved drugs is approximately 0.18, thus DL ≥ 0.18 was used as the screening threshold to retain compounds with drug-like structural features.

Target data for each compound were extracted from the TCMSP database. To enhance data comprehensiveness, the Batman-TCM database^21^ (http://bionet.ncpsb.org.cn/batman-tcm/index.php) was queried using the same keywords. The retrieved chemical components were cross-referenced with the PubChem database^22^ (https://pubchem.ncbi.nlm.nih.gov/) to obtain SMILES representations. The compound lists from both databases were merged, duplicates removed, and the final dataset was input into the SwissTargetPrediction database^23^ (https://swisstargetprediction.ch/) for target prediction. This tool ranks likely protein targets based on structural similarity, and the identified targets were standardized using the UniProt database^24^ (http://www.uniprot.org/).

#### Collection of disease targets for oligoasthenozoospermia

Potential targets for oligoasthenozoospermia were retrieved from the Online Mendelian Inheritance in Man (OMIM) database^25^ (https://omim.org/), the GeneCards database^26^, the DrugBank database^27^ (https://go.drugbank.com/), and the DisGeNET database^28^ (https://disgenet.com/) using the keywords “asthenospermia,” “oligoasthenozoospermia,” and “oligospermia.” The resulting targets were merged, deduplicated, and standardized using the UniProt database.

#### Identification of potential targets of ZGW for treating oligoasthenozoospermia

Drug-related and disease-related targets were analyzed using Venny 2.1.2^29^ (http://www.liuxiaoyuyuan.cn/) to identify overlapping targets that may contribute to ZGW’s therapeutic effects on oligoasthenozoospermia.

#### Construction of the common target protein–protein interaction network

Common targets were imported into the STRING database^30^ (https://cn.string-db.org/) with an interaction score threshold of > 0.9. The interaction network was visualized using Cytoscape 3.7.2^31^ to construct a protein–protein interaction (PPI) network and identify key targets.

#### Construction of the herb–compound–target network

A network comprising ZGW’s herbs, active compounds, and targets was constructed using Cytoscape 3.7.2. Key compounds were identified based on their degree values.

#### Functional and pathway enrichment analysis

Common targets of ZGW and oligoasthenozoospermia were analyzed using the Metascape database^32^ (https://metascape.org/gp/index.html), with the species set to Homo sapiens. Gene Ontology (GO) and Kyoto Encyclopedia of Genes and Genomes (KEGG) pathway enrichment analyses were performed with a significance threshold of *P* < 0.01. Visualization was conducted using the Bioinformatics platform^33^ (https://www.bioinformatics.com.cn/). KEGG Mapper^34^ (https://www.genome.jp/kegg/) was used to generate pathway diagrams for key signaling pathways.

### Molecular docking of key compounds and core targets

The top 10 targets, ranked by degree values, were retrieved from the UniProt database. Their protein structures were downloaded from the Protein Data Bank (PDB) in PDB format. The top four core compounds were selected based on degree values, and their 3D structures were obtained from the TCMSP database in MOL2 format.Protein and ligand structures were preprocessed using PyMOL^35^ before molecular docking was performed using AutoDockTools-1.5.7^36^. The docking results were visualized in PyMOL (3D) and LigPlot+ (2D)^37^.

### Molecular dynamics simulation

The protein–ligand complex with the lowest binding energy was subjected to a 100 ns molecular dynamics (MD) simulation using GROMACS 2023^38^. The CHARMM36^39^ force field was applied to the protein, while the GAFF2 force field was used for ligand topology. The system was solvated in a TIP3P water model within a cubic box under periodic boundary conditions^40^. Electrostatic interactions were handled using the particle mesh Ewald (PME) method, and the Verlet al.gorithm was applied for van der Waals interactions. The system underwent energy minimization followed by 100,000 steps of equilibration in both NVT (constant volume and temperature) and NPT (constant pressure and temperature) ensembles, with coupling constants of 0.1 ps and a duration of 100 ps. A cutoff distance of 1.0 nm was set for both van der Waals and Coulomb interactions. Finally, a 100 ns MD simulation was carried out at 300 K and 1 bar pressure using GROMACS 2023.

### Experimental validation

#### Drugs and reagents

Triptolide (GTW) tablets (10 mg/tablet, batch number: 130801) were purchased from Xinlonghai Pharmaceutical Co., Ltd., Anhui, China. ZGW (batch number: WX-ZL-13, 230805) was obtained from Zhongjing Wanxi Pharmaceutical Co., Ltd., Nanyang, China, in the form of water-honey pills, dissolved in warm water and stored at low temperature. Fetal bovine serum (10%) (catalog number: 04-001−1ACS) and high-glucose DMEM (catalog number: 06-1055-57-1ACS) were purchased from Biological Industries, Israel. Trizol reagent was purchased from Invitrogen, USA; M-MLV reverse transcription kit was obtained from Promega, USA; and real-time PCR amplification kit was supplied by Qiagen, USA. Absolute ethanol (CAS number: 64-17-5) and xylene (CAS number: 1330-20-7) were purchased from Sinopharm Chemical Reagent Co., Ltd., China. RIPA lysis buffer (strong) (G2002-100ML), BCA protein quantification kit (G2026-200T), 5× SDS-PAGE protein loading buffer (G2075-100ML), primary antibody diluent (G2025-100ML), Western secondary antibody diluent (G2009-100ML), and antibody elution buffer (G2009-100ML) were obtained from Servicebio, China. PBS buffer (ZH-0001), H.E staining kit (RY-0002), and DAB chromogenic reagent (ZH-0005) were supplied by Henan Chaoran Biotechnology Co., Ltd., China. TUNEL assay kit (11684817910) was purchased from Roche, Switzerland. TP53 antibody (ab08878), PKC antibody (ab179522), and NF-κB antibody (ab76302) were purchased from Abcam, UK.

#### Instruments

The instruments used in this study included a real-time PCR system (ABI 7500 Fast, ABI, USA), a constant voltage and current electrophoresis system (Bio-Rad, USA), a digital gel imaging system (BINTA, China), a computer image analysis system (Image-Pro Plus Analysis Software, USA), and a refrigerated high-speed centrifuge (Thermo MR 23i, Thermo, USA). Additional instruments included an electronic balance (Sartorius BS224S, Sartorius, Germany), a nucleic acid UV spectrophotometer (Biophotometer, Eppendorf, Germany), a vertical electrophoresis tank (Bio-Rad MP3, Bio-Rad, USA), a semi-dry transfer blot system (Bio-Rad Trans-Blot SD, Bio-Rad, USA), and a high-speed centrifuge (Eppendorf 5417 C, Eppendorf, Germany). Other equipment comprised a micropipette (Eppendorf Research plus, Eppendorf, Germany), an ultrasonic cell disruptor (JY92-11 N, Ningbo Xinzhi Biotechnology, China), a chemiluminescence imaging system (SCG-W3000, Servicebio, China), a magnetic stirrer (MS-150, Servicebio, China), a thermostatic water bath (W20M-2, SHELLAB, USA), a liquid nitrogen storage tank (YDS-30–125, Dongya, China), an optical microscope (L340099, Olympus, Japan), a transmission electron microscope (H-7500, Hitachi, Japan), and an inverted microscope (Ci-S, Nikon, Japan). All instruments were high-precision laboratory equipment, ensuring the reliability and accuracy of experimental data.

#### In *vitro* experiments

The GC-1spg mouse spermatogonia cell line (Procell Life Technology, Catalog No. CL-0600) was used to investigate GTW-induced damage and the protective effects of ZGW intervention. Cells were cultured in DMEM medium supplemented with 10% fetal bovine serum and antibiotics until they reached 70–80% confluence before treatment. Cells were divided into three groups: the control group (200 µL blank serum + 1.8 mL basal medium), the GTW group (200 µL GTW serum + 1.8 mL basal medium), and the ZGW group (200 µL GTW serum + 1.8 mL basal medium), and incubated for 24 h. After 24 h of initial culture, the medium was replaced with the corresponding treatment medium: control group received 200 µL blank serum + 1.8 mL basal medium; GTW group received 200 µL blank serum + 1.8 mL basal medium; ZGW group received 200 µL ZGW serum + 1.8 mL basal medium. Experimental sera were derived from animal experiments by collecting blood from the abdominal aorta, centrifuging, inactivating, filtering, and storing at − 80 °C for subsequent use.

#### CCK-8 assay

Cells were inoculated into a 96-well plate at 100 µL per well and pre-cultured in an incubator for 24 h. Subsequently, 10 µL of serum from the control, model and ZGW groups was added to each well (with six replicates per group), followed by a 24-hour incubation. After incubation, 10 µL of CCK-8 solution was added to each well (avoiding air bubbles that could interfere with absorbance detection), and the plate was incubated for an additional 1–4 h. The optical density (OD) at 450 nm was measured using a microplate reader. Cell viability was calculated using the normal cell group as the reference (100%).

#### Western blot (WB) in *vitro* experiments

After washing cells with PBS, suspended cells were collected by centrifugation at 2000 rpm, 4 °C for 5 min, while adherent cells were scraped after PBS washing. Cells were lysed in RIPA buffer containing protease inhibitors on ice for 30 min, followed by centrifugation at 12,000 rpm, 4 °C for 10 min. The supernatant was collected as the total protein. Protein concentration was determined using a BCA kit, and samples were denatured by boiling in loading buffer. Proteins were separated by SDS-PAGE, transferred onto a PVDF membrane, blocked with 5% skim milk for 30 min, and incubated overnight at 4 °C with primary antibodies (NF-κB, PKC, TP53, 1:2000). Afterward, membranes were incubated with HRP-conjugated secondary antibodies (1:2000) at room temperature for 1 h. Protein bands were detected using ECL, and band intensity was analyzed with AIWBwellTM software. GAPDH was used as an internal control.

#### Real-time quantitative PCR (RT-qPCR) in *vitro* experiments

Cells were washed with PBS, lysed with Trizol, and incubated at room temperature for 5 min. RNA was extracted using chloroform, followed by emulsification by vortexing and centrifugation at 12,000 ×g, 4 °C for 15 min. The aqueous phase was collected, and RNA was precipitated with isopropanol, centrifuged at 12,000 ×g, 4 °C for 10 min, and washed with 75% ethanol. The RNA pellet was dried and dissolved in DEPC-treated water. RNA concentration was determined, and cDNA was synthesized using the M-MLV reverse transcription kit. The qPCR reaction system included cDNA, primers, and SYBR mix. Thermal cycling conditions were 94 °C for 2 min, 45 cycles of 94 °C for 5 s, 60 °C for 30 s, and a final extension at 72 °C for 10 min. Relative gene expression was calculated using the 2-∆∆CT method, with GAPDH as an internal control. Primer sequences are listed in Table 1.

**Table 1:** List of primer sequences for real-time quantitative PCR for in *vivo* experiments.

Gene	Forward primers (5’−3’)	Reverse primer (5’−3’)
NF-κB	GGAGAAGGCTGGAGAAGATG	GCTCATACGGTTTCCCATTT
PKC	AGGAAGCCGTCTCTATCTGC	TTGGGCTTGAATGGTGGTTG
TP53	AAACGCTTCGAGATGTTCCG	GTAGACTGGCCCTTCTTGGT
GAPDH	GGTGAAGGTCGGTGTGAACG	CTCGCTCCTGGAAGATGGTG

#### In *vivo* experiments

##### Ethics statement

This study was conducted in accordance with the ARRIVE guidelines. All experimental procedures involving animals were performed in compliance with relevant institutional, national, and international guidelines and regulations. The animal protocol was reviewed and approved by the Experimental Animal Welfare Ethics Committee of the First Affiliated Hospital of Henan University of Traditional Chinese Medicine (Approval No. YFYDW2023033), in accordance with the Guidelines for the Care and Use of Laboratory Animals.

##### Construction of the OAS rat model

Based on the in vitro findings, we further conducted animal experiments to evaluate the protective effects of ZGW against GTW-induced reproductive injury. A total of 42 eight-week-old male Sprague–Dawley rats (200–220 g; Zhengzhou University Experimental Animal Center, license SCXK (Jing) 2019-0004) were housed under controlled conditions (18–25 °C, 50–80% humidity) with free access to food and water and acclimatized for one week. Rats were randomly assigned to a blank control group (*n* = 15) and a GTW group (*n* = 27). GTW was administered by gavage at a dose of 40 mg/kg/day for 4 weeks, a regimen widely reported to induce testicular and spermatogenic dysfunction in rodent models, while controls received an equal volume of distilled water. At the end of week 4, three rats per group were sacrificed to validate successful model establishment (sperm concentration and motility), and these animals were excluded from subsequent analyses. The remaining rats were allocated to three cohorts for the main experiment: control (*n* = 12), persistent-model (*n* = 12), and ZGW-treated (*n* = 12).

##### Pharmacological intervention

From the 5th to the 8th week, Groups A and B received daily gavage of deionized water (10 ml/kg), while Group C received daily gavage of ZGW suspension at a dose of 1.89 g/kg (These doses were determined using the equivalent dose conversion factor based on body surface area, as determined to be optimal in preliminary experiments^13^.The selected doses of both GTW and ZGW are biologically relevant and have been supported by previous studies demonstrating their efficacy in modeling reproductive toxicity and evaluating the protective effects of traditional Chinese medicine formulations^42^.

##### Sample collection and processing

After 4 weeks of intragastric administration, all rats were fasted for 12 h and weighed. They were then anesthetized with 3% pentobarbital sodium (30 mg/kg, intraperitoneally). Blood samples were collected via the abdominal aorta, centrifuged at 3,000 rpm for 10 min to isolate serum, which was subsequently heat-inactivated at 56 °C for 30 min, filtered through a 0.22 μm membrane to remove bacteria, and stored at − 80 °C for future analysis. The testes and epididymides were also harvested for subsequent experiments.

At the end of the experiment, all animals were euthanized by cervical dislocation under deep anesthesia using 3% pentobarbital sodium (30 mg/kg, intraperitoneally).

##### Histological and ultrastructural observation of testicular tissue

A 1 mm³ sample of the right testicular tissue from each rat was fixed with 2.5% glutaraldehyde and 1% osmium tetroxide. Transmission electron microscopy (TEM) was used to observe the ultrastructure of spermatogenic cells, seminiferous tubules, convoluted seminiferous tubules, and capillary endothelial cells, focusing on mitochondria, nuclei, and the Golgi apparatus^43^.

##### Hematoxylin and eosin (HE) staining

Tissue samples were fixed in 10% formalin for 24–48 h, followed by gradient ethanol dehydration (70%–100%) and xylene clearing. The tissues were then embedded in molten paraffin and sectioned into 4 μm thick slices. After deparaffinization with xylene, the sections were rehydrated using a graded ethanol series. Subsequently, the sections were immersed in hematoxylin staining solution for 5–10 min, differentiated, and then blued with ammonia water. Next, they were immersed in eosin staining solution for 1–3 min, rinsed, and sequentially dehydrated, cleared, and mounted. Finally, optical microscopy was used for observation, showing nuclei stained blue-purple and cytoplasm stained pink^44^.

##### TUNEL assay for spermatogenic cell apoptosis

The left testes of rats were collected, fixed in 4% paraformaldehyde for 24 h, and cut into tissue blocks of approximately 3 mm. After gradient ethanol dehydration (75%, 85%, 90%, 95%, and 100% ethanol for 1 h each), xylene clearing, and paraffin embedding, tissues were sectioned at 4 μm thickness and mounted on glass slides. Sections were baked at 65 °C for 1 h before staining.

The sections were dewaxed, rehydrated through graded ethanol, and treated with proteinase K (20 µg/mL) at 37 °C for 20 min to increase permeability. Endogenous peroxidase activity was quenched with 3% H₂O₂ for 10 min at room temperature. The sections were then incubated with the TUNEL reaction mixture (In Situ Cell Death Detection Kit, POD, Roche, Germany) at 37 °C for 1 h in a humidified chamber, followed by incubation with converter-POD solution at 37 °C for 30 min. Apoptotic nuclei were visualized with 3,3′-diaminobenzidine (DAB, Beijing Zhongshan Jinqiao Biological Co., Ltd., China), producing a brown signal, and counterstained with hematoxylin. After dehydration and mounting with neutral resin, sections were ready for observation^45^.

##### WB in *vitro* experiments

After tissue homogenization, the samples were lysed on ice for 30 min, centrifuged at 12,000 rpm at 4 °C for 10 min, and the supernatant was collected. Protein quantification, SDS-PAGE electrophoresis, and Western Blot analysis were performed following the same protocols used in the in *vitro* experiments.

##### RT-qPCR in *vitro* experiments

RNA extraction from tissues was performed as described in the in *vitro* experiments, with RNA purity and concentration measured using a UV spectrophotometer. The qPCR reaction was carried out using the SYBR kit (Qiagen) on an ABI 7500 Fast system, with each sample run in triplicate. Primer sequences are listed in Table 2, and primers were synthesized by Sangon Biotech (Shanghai). Data analysis followed the same methods used in the in *vitro* experiments.

**Table 2:** List of primer sequences for real-time quantitative PCR in *vitro* experiments.

Gene	Forward primers (5’−3’)	Reverse primer (5’−3’)
NF-κB	GCGGCAGAAGTTTGAGAGAG	GTCCCTGTTGCCATTGTTGT
PKC	TCCGAGTAGACAGCATCACC	AGGAGCATAGGAAGCACTCC
TP53	GCGGTCTCCTCGAACTCTTT	CATCGAGCTCCCTCTGAGTC
GAPDH	GCTTCCTCTGGGCCTTCTAA	CAACTCCCTCAAGATTGTCAGCAA

#### Statistical analysis

All experiments were independently repeated at least three times, and data are expressed as the mean ± standard deviation. Statistical analysis was performed using GraphPad Prism (version 10.0.0). All measurement data are presented as the mean ± standard deviation. If the data followed a normal distribution, one-way ANOVA was used to compare multiple groups. For equal variances, the Student-Newman-Keuls test and the Least Significant Difference test were used for multiple comparisons. If the variances were unequal, Welch’s test and the Games-Howell test were applied for multiple comparisons. For data that did not meet normality, non-parametric tests were used. A p-value < 0.05 was considered statistically significant, and a p-value < 0.01 was considered highly significant.

## Results

### Acquisition of intersection targets

The OMIM, GeneCards, DisGeNET, and DrugBank databases were queried, yielding 74, 1004, 236, and 1 related genes, respectively. After analyzing the gene targets and removing duplicates, a total of 1119 disease targets associated with OAS were identified.

**Fig. 2:**
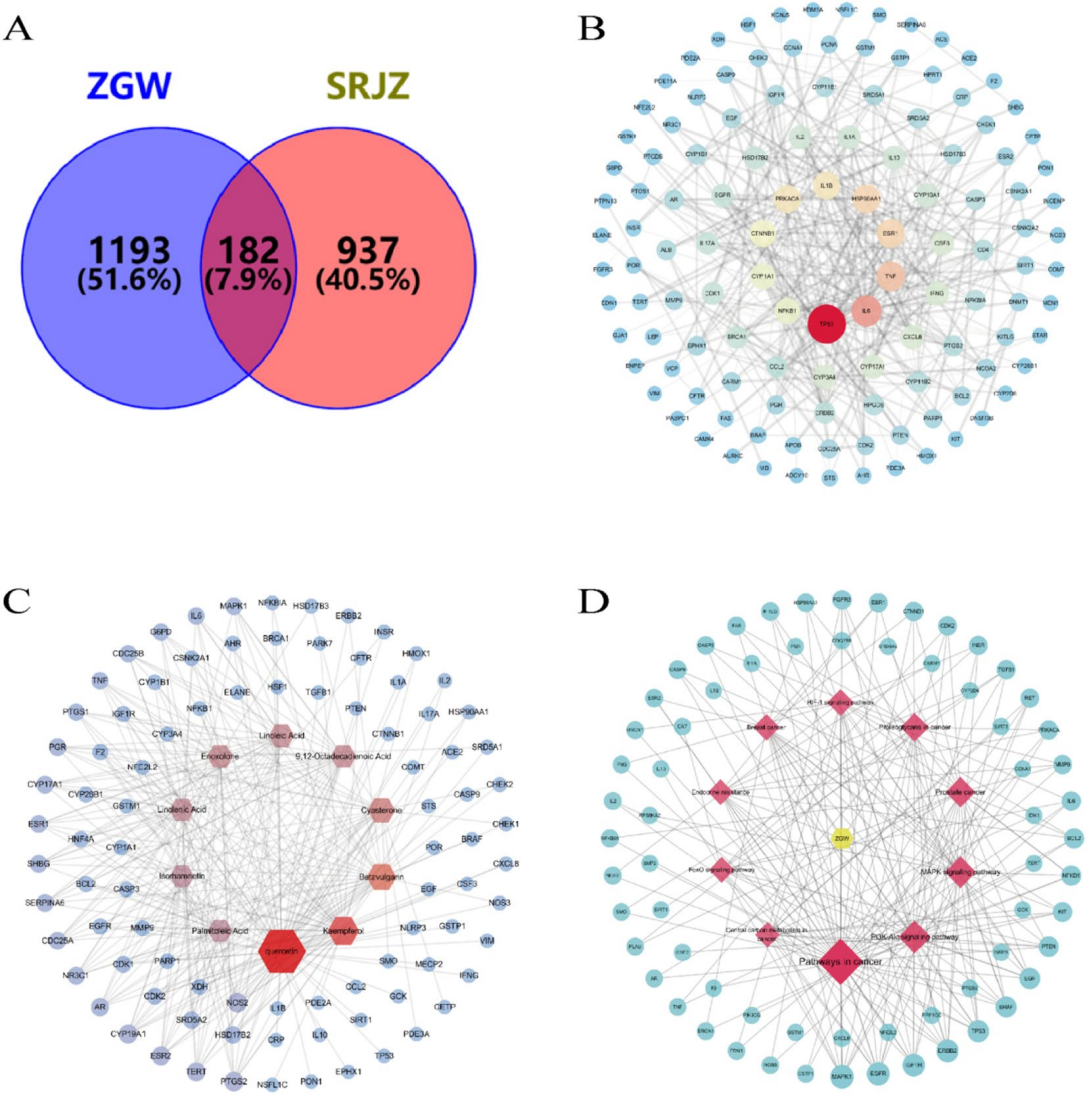
The intersection targets, PPI network, component-target network, and drug-pathway-target network of ZGW in the treatment of OAS.(A) Venn diagram of the intersection targets of ZGW in the treatment of OAS.(B) PPI network construction of ZGW in the treatment of OAS.(C) Core component-target network of ZGW in the treatment of OAS.(D) Drug-pathway-target network of ZGW in the treatment of OAS.

The active components of ZGW were collected, and duplicates were removed, resulting in 307 components and 1375 related targets. The drug targets and disease targets were imported into Venny 2.1, which identified 182 intersecting targets, as shown in Fig. 2A.

### Construction of the intersection target interaction network

The 182 intersecting targets were imported into the STRING database, and their interaction relationships were analyzed using Cytoscape 3.7.2 software. A network with 122 nodes and 345 edges was constructed, as shown in Fig. 2B. The CytoNCA function was used to assess the degree, betweenness centrality, and closeness centrality of the nodes, and the top 10 targets were selected as core targets.

### Single herb-component-target network construction

The drug and target data were imported into Cytoscape 3.7.2 software, and the CytoNCA function was used to identify core components. The top 10 components, ranked by degree value, were selected to create the component-target network, as shown in Fig. 2C. This network involved 100 nodes and 278 edges. The core components are listed in Table 3.

**Table 3:** Core components of the top ten degree rankings of Zuogui pills.

Degree	Chemical compound	Herbal medicine
64	Quercetin	CNX GQZ SZY TSZ
33	Kaempferol	SZY TSZ
29	Betavulgarin	CNX
25	Cyasterone	CNX
22	9,12-Octadecadienoic Acid	SDH GQZ SZY
22	Enoxolone	GBJ LJJ
22	Linoleic Acid	SDH GQZ SZY
21	Linolenic Acid	SZY
20	Isorhamnetin	SZY TSZ
20	Palmitoleic Acid	SDH SZY

### Biological function and pathway analysis

**Fig. 3:**
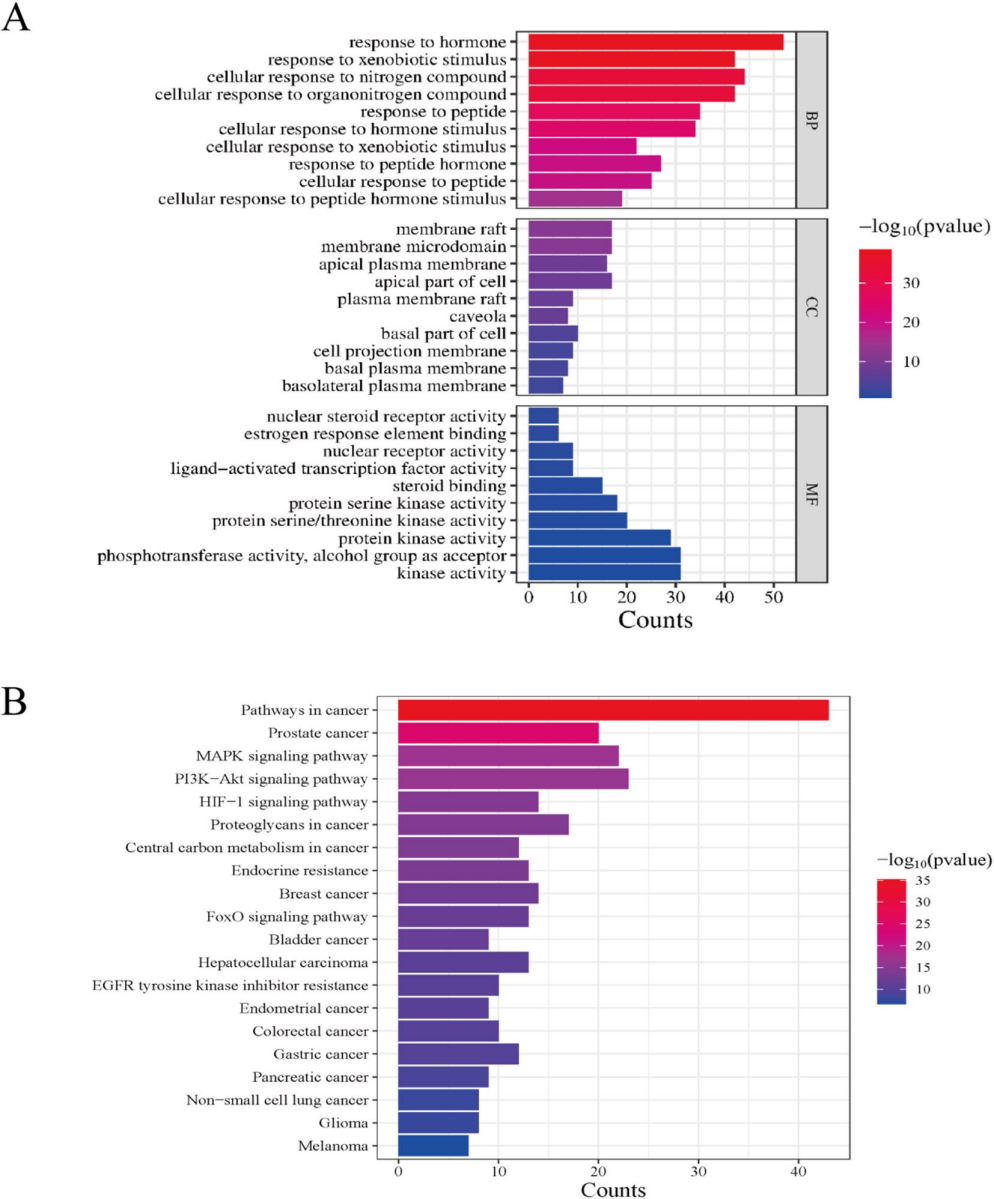
GO, KEGG enrichment analysis.(**A**) GO enrichment analysis of ZGW in the treatment of OAS: Biological Process (BP), Cellular Component (CC), and Molecular Function (MF).(**B**) KEGG enrichment analysis of ZGW in the treatment of OAS.

GO analysis revealed that the 182 protein targets involved in ZGW treatment for OAS were subjected to GO and KEGG pathway analysis. The top 10 most enriched Biological Process (BP), Cellular Component (CC), and Molecular Function (MF) terms are shown in Fig. 3A. Many enriched GO terms were related to hormone responses, such as “response to hormone” and “cellular response to hormone stimulus,” suggesting that these genes are closely associated with hormonal regulation and may play important roles in signal transduction and stress responses. Other terms involved responses to peptides and nitrogen compounds, such as “response to peptide” and “cellular response to nitrogen compound,” indicating that the genes may regulate cellular responses to external stimuli. Several terms referred to specific cellular locations, such as “membrane raft,” “apical plasma membrane,” and “basal part of cell,” highlighting the significance of these gene products in cell membranes and membrane-related structures. Structures such as “caveola” and “membrane raft” have been implicated in functions such as signal transduction and material transport, suggesting that core targets may be involved in these cellular functions.Terms like “protein kinase activity” and “protein serine/threonine kinase activity” suggest that core targets may have protein kinase activity and be involved in signal transduction. The terms “steroid binding” and “nuclear receptor activity” imply that these genes may play a role in hormone binding and nuclear receptor regulation, indicating a potential link to hormone signaling pathways.From the GO enrichment analysis, it is evident that this gene set is significantly enriched in hormone response, cell membrane structures, and kinase activity, likely playing a crucial role in signal transduction, cellular stress responses, and hormone regulation.

**Fig. 4:**
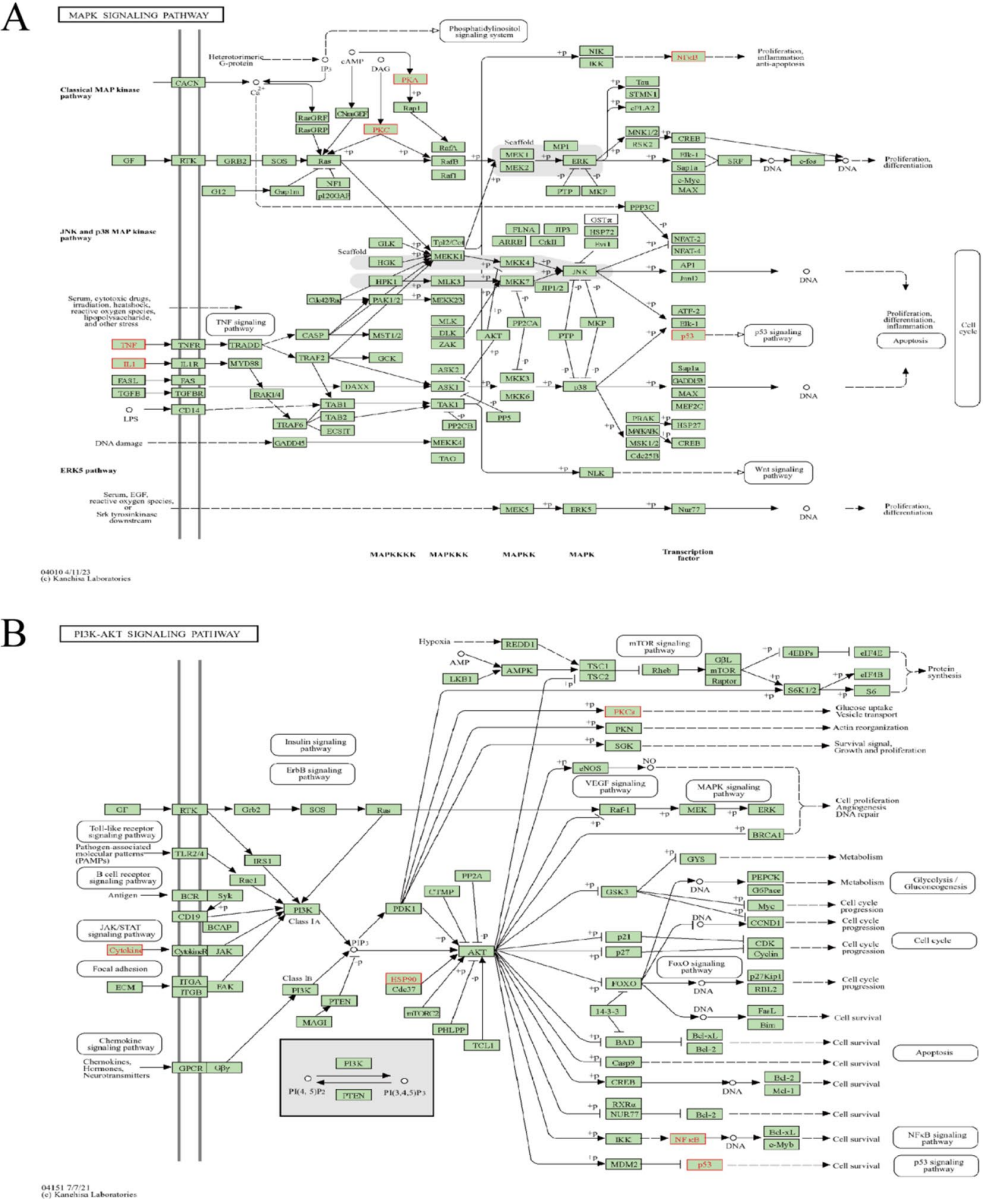
pathway diagrams.(**A**) MAPK signaling pathway diagram of ZGW in the treatment of OAS.(**B**) PI3K-AKT signaling pathway diagram of ZGW in the treatment of OAS.

In KEGG enrichment analysis, the top 10 pathways were selected for enrichment analysis and presented as a bubble plot in Fig. 3B. A network diagram of the pathways, related genes, and corresponding components was constructed, as shown in Fig. 2D, with 77 nodes and 201 edges. Major pathways included cancer pathways, PI3K-AKT signaling, MAPK signaling, and HIF-1 signaling, all of which have a positive impact on metabolic and endocrine regulation, sperm survival, and overall reproductive health. The significant enrichment of the PI3K-Akt, MAPK, and HIF-1 signaling pathways (with very low p-values) and the high GeneRatio suggest that ZGW may regulate cell growth and survival through these three classic signaling pathways, which are especially important for spermatogenesis and sperm survival. KEGG pathway diagrams for PI3K-AKT and MAPK signaling are shown in Fig. 4A and B, highlighting their association with TP53, PKC, and NF-κB, which are central to the current study.

### Molecular docking results for components-targets

**Fig. 5:**
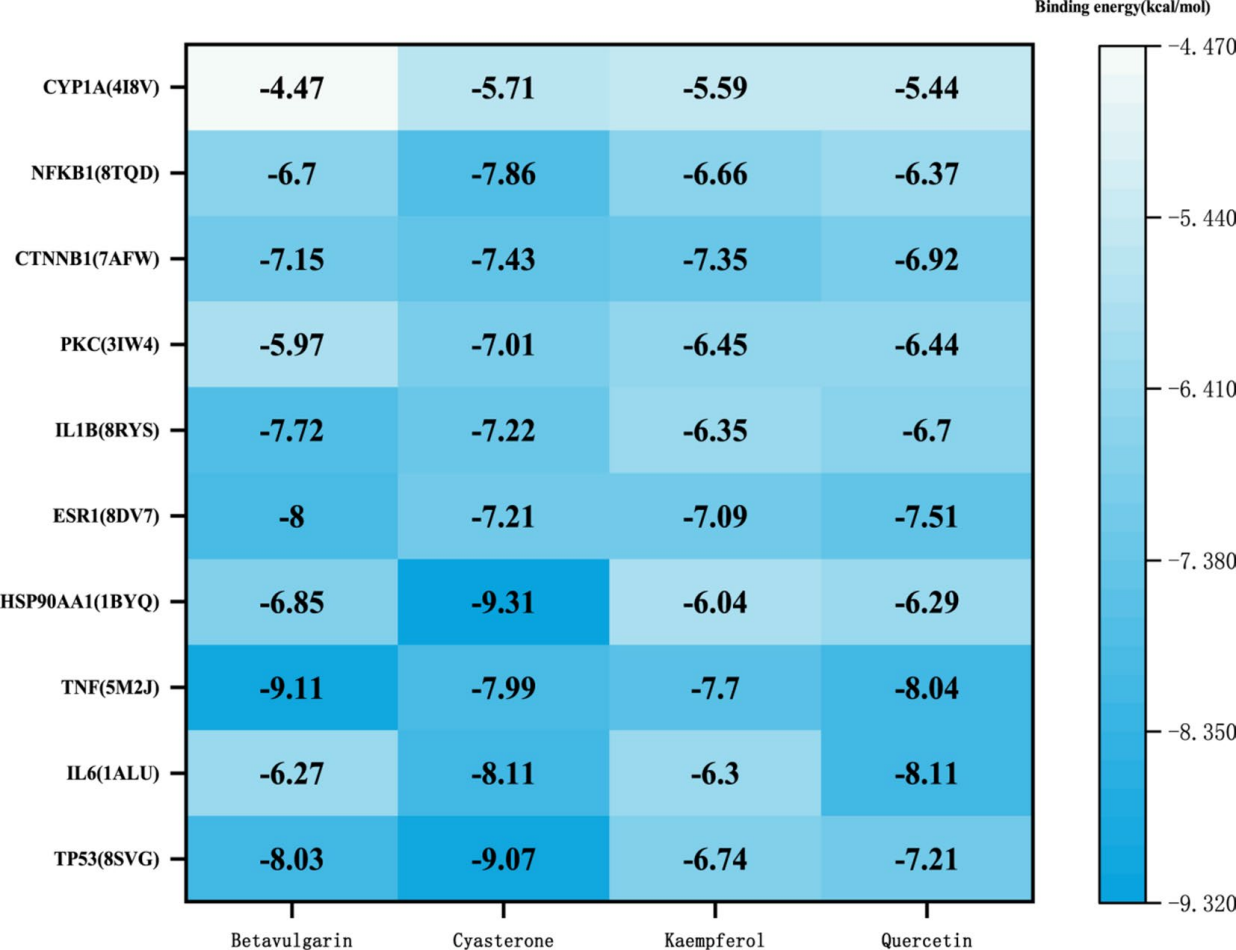
Binding energies of key active components to core proteins.

**Fig. 6:**
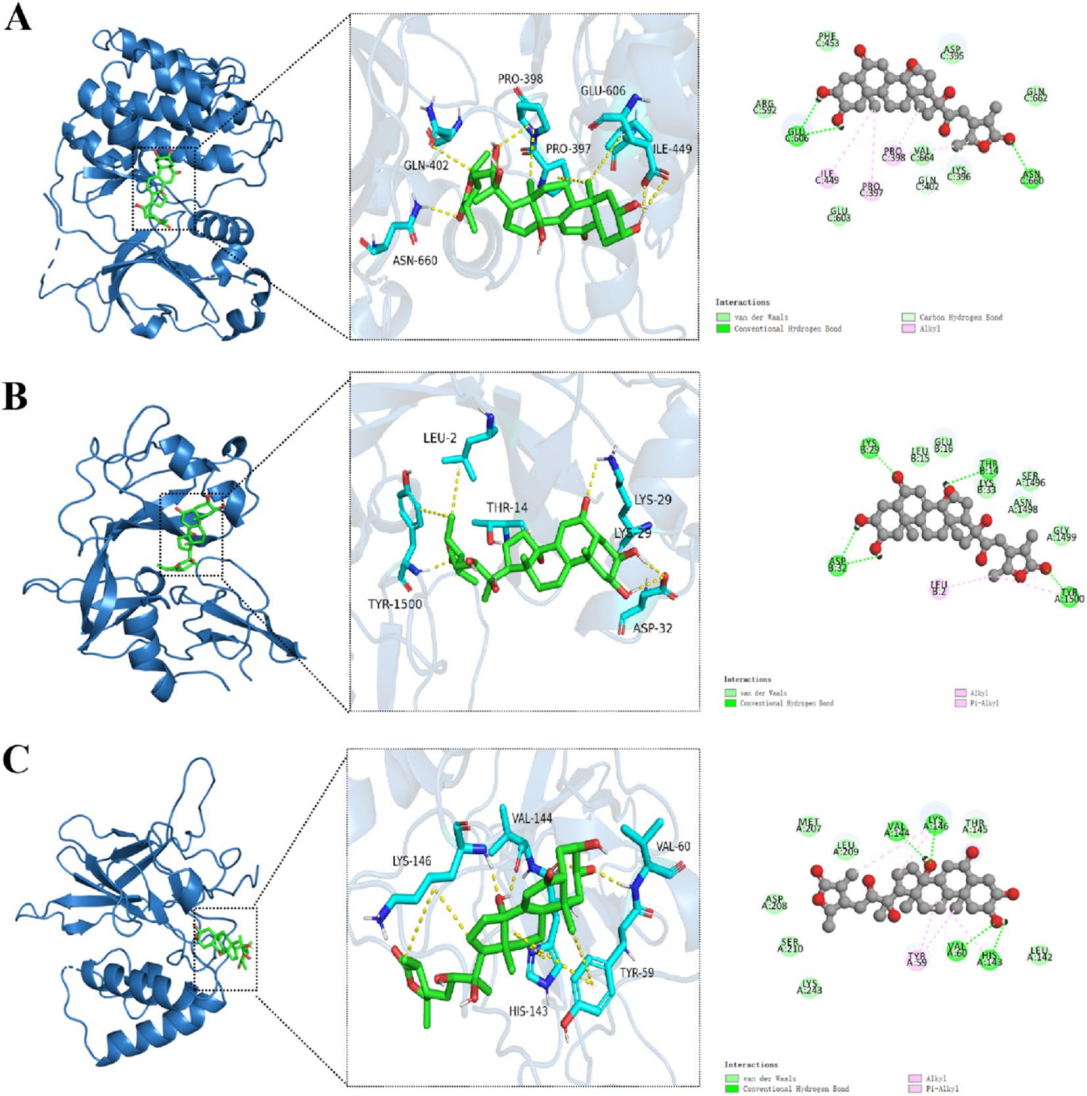
Molecular docking visualization of key compounds and core target proteins.(**A**) Molecular docking visualization of Cyasterone with PKC (3IW4).(**B**) Molecular docking visualization of Cyasterone with TP53 (8SVG).(**C**) Molecular docking visualization of Cyasterone with NF-kB (8TQD).

From the ZGW active component-target network, the top 10 compounds with the highest degree values were selected as ligands. Their three-dimensional structures were retrieved, and core targets from the drug-disease common target PPI network and major pathway-target networks were selected as receptors. After selecting appropriate target proteins from the PDB, their PDB formats were downloaded and imported into AutoDock for molecular docking. Polar hydrogen atoms, charges, and grid box sizes were adjusted before generating the docking files. AutoDock was used to dock the core targets with the ligands, and the docking results with the lowest binding energy were visualized using PyMOL. Binding energies lower than − 5 kcal/mol indicate strong affinity, while energies lower than − 7 kcal/mol suggest extremely strong affinity. The docking results were visualized using PyMOL and plotted using Origin software, as shown in Fig. 5. The lowest binding energy docking results for TP53, PKC, and NF-κB were selected for further visualization in Fig. 6.

From Fig. 6, it can be seen that Cyasterone forms hydrogen bonds with amino acid residues GLU606, ASN660 on PKC (3IW4), and alkyl bonds with PRO397, ILE449, PRO398. It also interacts with ASP395, PHE453, ARG592, GLU603, LYS396, and VAL664 via van der Waals forces, as shown in Fig. 6A. Cyasterone also forms hydrogen bonds with LYS29, ASP32, THR14, and TYR1500 on TP53 (8SVG), and forms alkyl and Pi-alkyl bonds with additional residues (Fig. 6B). On NF-κB (8TQD), Cyasterone forms hydrogen bonds with LYS146, VAL60, VAL144, HIS143, and alkyl interactions with LYS146 and TYR59, with additional van der Waals interactions with LEU209, MET207, ASP208, and others, as shown in Fig. 6C. These results suggest that Cyasterone from ZGW has a strong binding affinity with TP53, PKC, and NF-κB.

### Molecular dynamics analysis of key components-targets

**Fig. 7:**
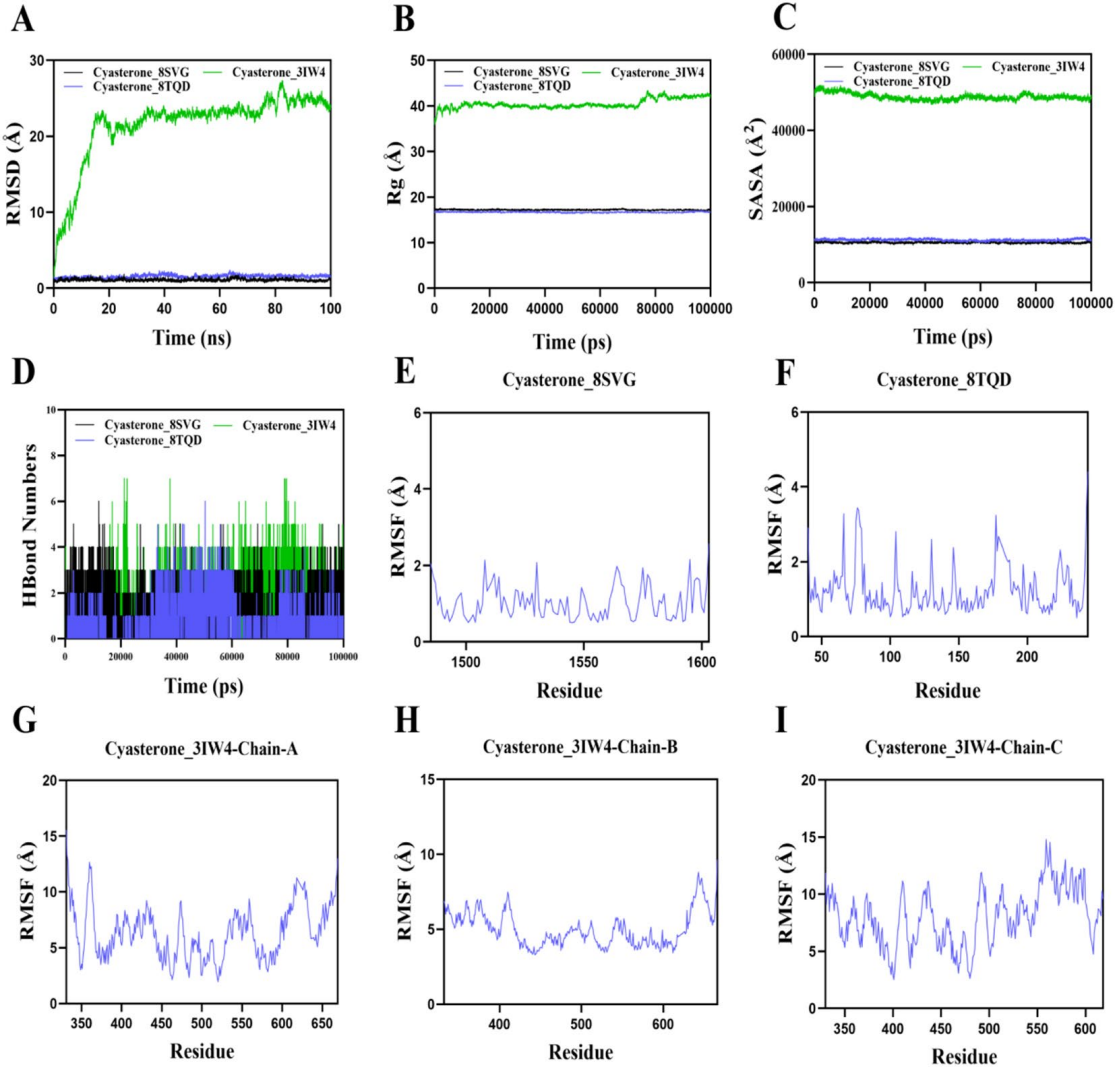
Molecular dynamics simulation of key compounds and core target proteins.(**A**) Root Mean Square Deviation (RMSD) analysis.(**B**) Radius of Gyration (Rg) analysis.(**C**) Solvent Accessible Surface Area (SASA) analysis.(**D**) Hydrogen Bond Numbers (HBond Numbers) analysis.(**E**-**I**) Root Mean Square Fluctuation (RMSF) analysis.

Root Mean Square Deviation (RMSD) is an effective indicator of the stability of protein-ligand complexes, reflecting the deviation of atomic positions from their initial configuration. A smaller deviation signifies greater stability. As shown in Fig. 7A, the Cyasterone_3IW4 complex reached equilibrium after 35 ns and fluctuated around 22 Å, indicating instability in biological environments. To further differentiate between Cyasterone_8SVG and Cyasterone_8TQD, a comparison of their molecular dynamics was performed (Supplementary Fig. 1). Both complexes reached equilibrium after 10 ns, with fluctuations of 0.9 Å and 1.1 Å, respectively. The Cyasterone_8SVG complex exhibited the lowest RMSD, indicating higher stability.

Further analysis of the Radius of Gyration (Rg) and Solvent Accessible Surface Area (SASA) values for the Cyasterone_8SVG complex showed stability throughout the simulation, suggesting that the target protein-ligand complex maintained a compact and stable structure (Fig. 7B and C, Supplementary Figs. 1B and 1 C).

Hydrogen bonds play a critical role in ligand-protein binding. The number of hydrogen bonds formed during the dynamics process between the small molecules and target proteins is shown in Fig. 7D and Supplementary Fig. 1D. The number of hydrogen bonds for the Cyasterone_8SVG complex ranged from 0 to 6, with approximately 3 hydrogen bonds on average, indicating a strong hydrogen bonding interaction between Cyasterone and the 8SVG target protein.

Root Mean Square Fluctuation (RMSF) reflects the flexibility of amino acid residues in a protein. As shown in Fig. 7E and I, the RMSF values of the Cyasterone_8SVG complex were relatively low (mostly below 2 Å), indicating low flexibility and high stability.

In conclusion, the Cyasterone_8SVG complex demonstrated stable binding and favorable hydrogen bond interactions, making Cyasterone a promising candidate for binding with the 8SVG target protein.

### In *vitro* cellular protective effects of ZGW

**Fig. 8:**
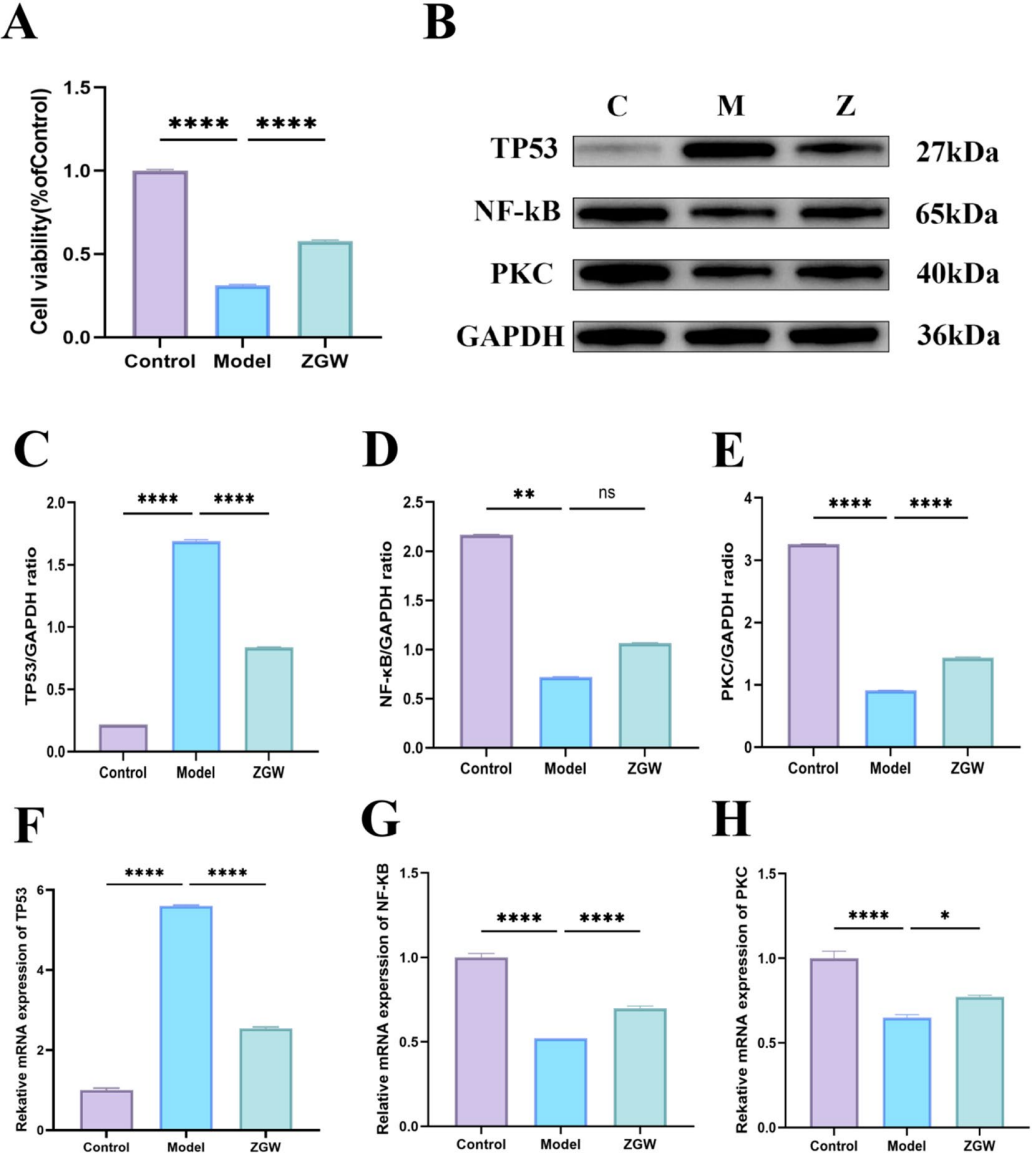
In *vitro* experimental results.(**A**) CCK-8 analysis of ZGW treatment for OAS.(**B**) WB analysis of OAS-related proteins after ZGW treatment.C: control group, M : model group, and Z : Zuo Gui Wan group.(**C**) TP53 protein expression in each group.(**D**) NF-κB protein expression in each group.(**E**) PKC protein expression in each group.(**F**) TP53 mRNA expression in each group.(**G**) NF-κB mRNA expression in each group.(**H**) PKC mRNA expression in each group.ns *P* > 0.05; ** *P* ≤ 0.01; **** *P* ≤ 0.0001.

Compared with the normal group, the model group exhibited significantly lower cell viability (*P* < 0.01). In contrast, the ZGW group showed a significant increase in cell viability compared to the model group (*P* < 0.01), as shown in Fig. 8A.

### WB and Q-PCR results in *vitro*

Western blot results (Figs. 7B-E) indicated significant differences in the expression levels of TP53, NF-κB, and PKC in rat testicular tissues across the groups. TP53 expression was significantly elevated in the model group and decreased after ZGW treatment. The expression levels of NF-κB and PKC were highest in the control group, followed by the ZGW group, and lowest in the model group. Q-PCR results (Figs. 7F-H) further validated the Western blot findings. TP53 expression in the ZGW group was slightly higher than in the blank control group but significantly lower than in the model group. NF-κB and PKC expression in the ZGW group was slightly higher than in the blank control group and significantly higher than in the model group. In *vitro* experiments preliminarily verified the regulatory effect of ZGW on TP53, NF-kB, and PKC expression, suggesting that it may exert therapeutic effects by modulating the expression of these proteins.

### Model validation

In the cell experiments, GTW serum significantly damaged GC-1spg cells, whereas ZGW serum partially alleviated this damage. Based on these results, we proceeded with animal experiments to further validate the protective effects of ZGW in vivo. After 4 weeks of GTW gavage, we assessed sperm concentration and motility to confirm the successful induction of OAS. Compared with the blank control group, GTW-treated rats exhibited a marked reduction in both parameters. Specifically, sperm concentration decreased from (59.0 ± 7.42) × 10^6/mL in controls to (16.91 ± 3.29) × 10^6/mL in the model group (*p* < 0.001), and progressive motility (PR + NP) declined from (68.31 ± 10.82)% to (14.88 ± 0.82)% (*p* < 0.001). These results verified the successful establishment of the OAS rat model (Supplementary Fig. 2).

### Ultrastructural observation of testicular tissue

**Fig. 9:**
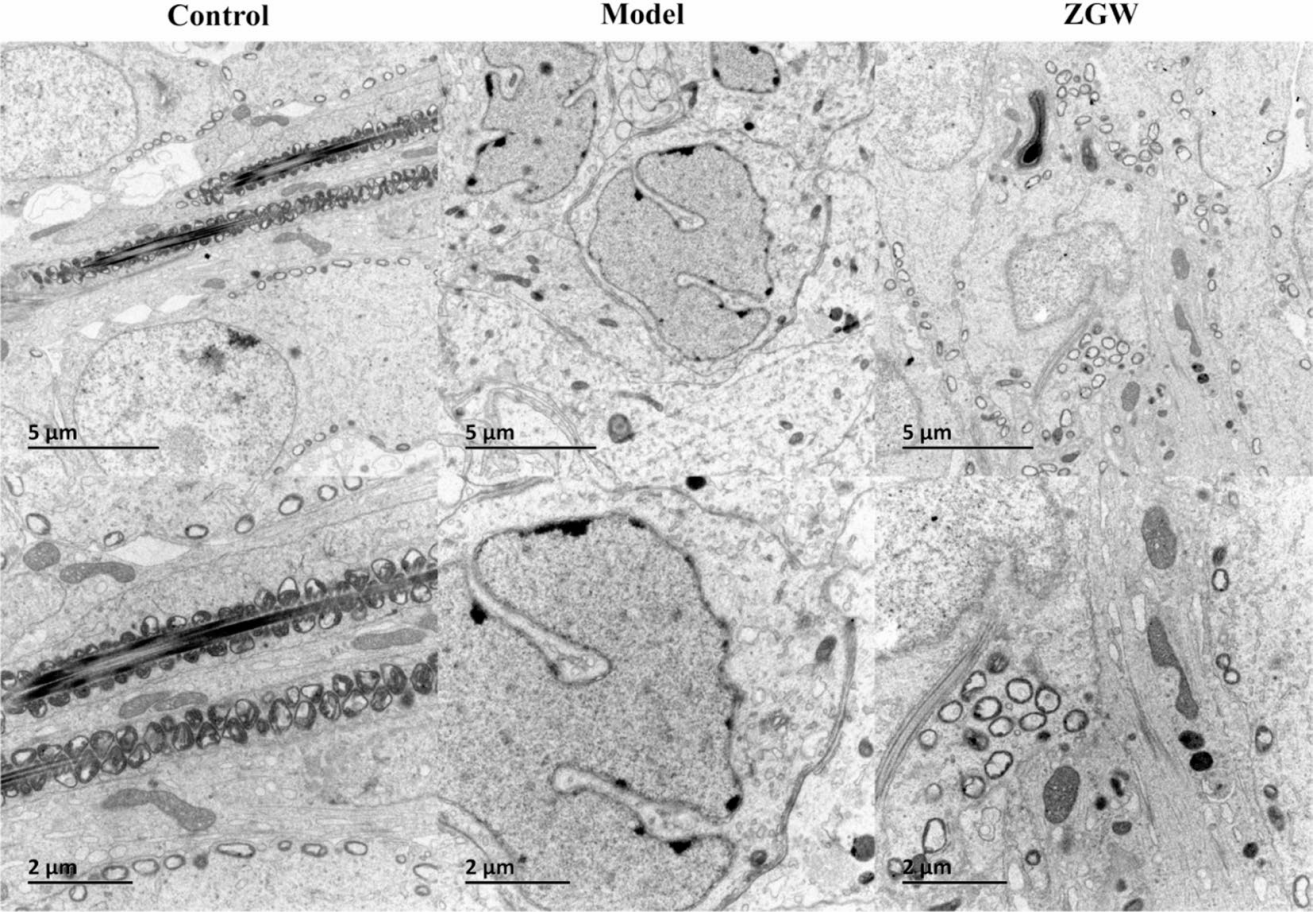
Comparative analysis of the ultrastructure of rat testicular tissues under Control, Model, and ZGW conditions.In the Control group, spermatocytes at various stages and cellular organelles appear normal, with a large number of sperm.In the Model group, the damage is severe, with swelling of cells and mitochondria, decreased sperm quantity, and the absence of mature sperm.In the ZGW group, the number of sperm and mitochondria is reduced compared to the normal condition.

In the control group, the morphology and structure of spermatogenic cells and organelles appeared normal, with abundant mitochondria, a well-formed and round cell nucleus, and a clearly visible nucleolus. The endoplasmic reticulum was distinctly observed, with autophagosomes present in varying quantities. In the model group, significant cellular damage and mitochondrial swelling were evident, including compromised cell membranes, altered nuclear shape (round or oval), pronounced endoplasmic reticulum expansion, and numerous autophagosomes. In the ZGW group, spermatogenic cell morphology and structure remained relatively intact. Although the number of mitochondria was slightly reduced compared to the control group, the nuclear structure was well-preserved, the nucleolus was clearly visible, and the endoplasmic reticulum was distinctly observed, with autophagosomes appearing in varying amounts (Fig. 9). These results strongly suggest that ZGW exerts a protective effect on damaged testicular tissue, as evidenced by the alleviation of extensive cellular damage in the ZGW group, where cellular structures remained more intact. This highlights ZGW’s role in preserving cellular integrity.

### Pathological morphological changes in testicular tissue

**Fig. 10:**
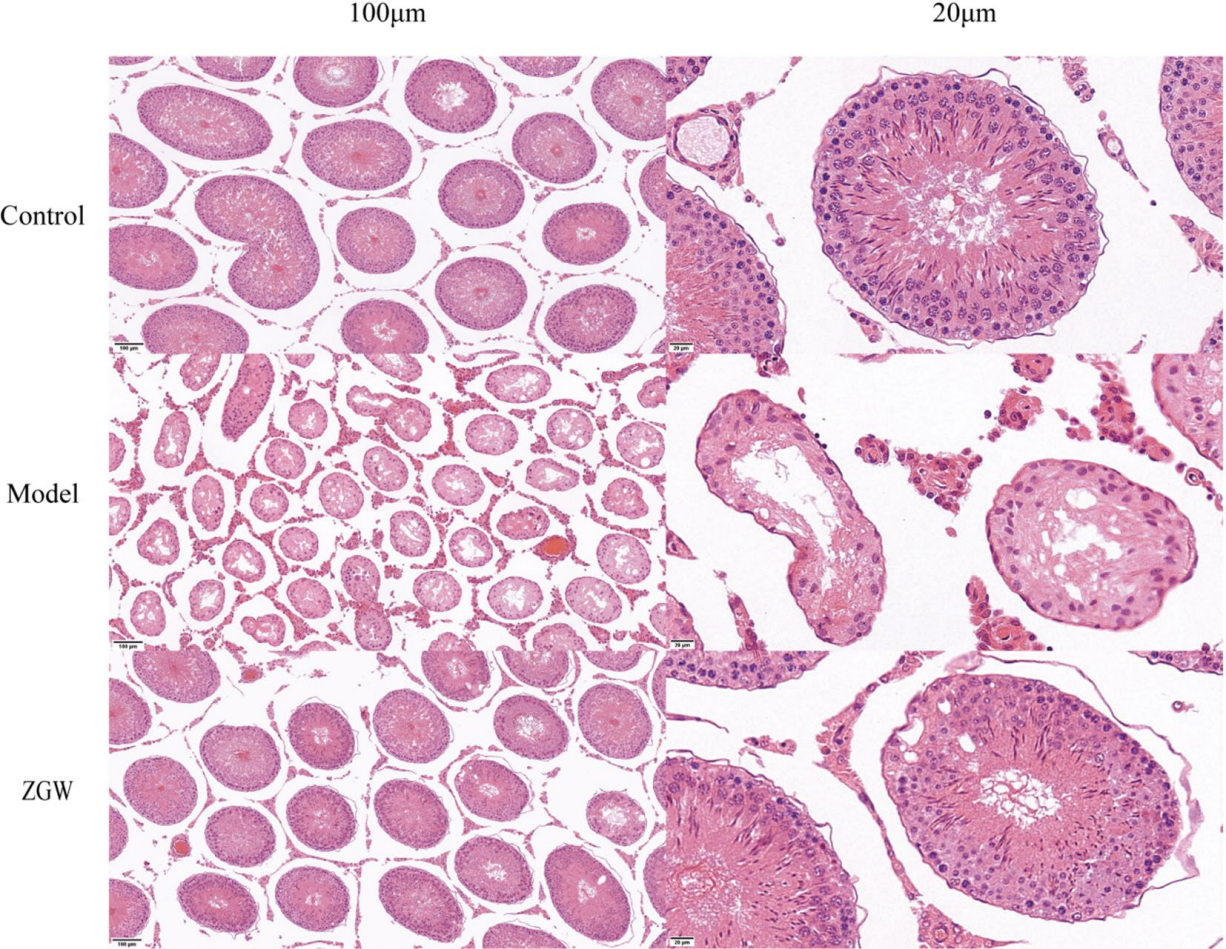
HE staining shows the protective effect of ZGW on rat testicular tissue.

HE staining of testicular tissue morphology (Fig. 10) revealed that, in the control group, the seminiferous tubules exhibited normal structure, were well-arranged, and contained a large number of morphologically normal sperm and spermatogenic cells at various developmental stages. The cell layers were intact and orderly, with no significant morphological abnormalities.

**Fig. 11:**
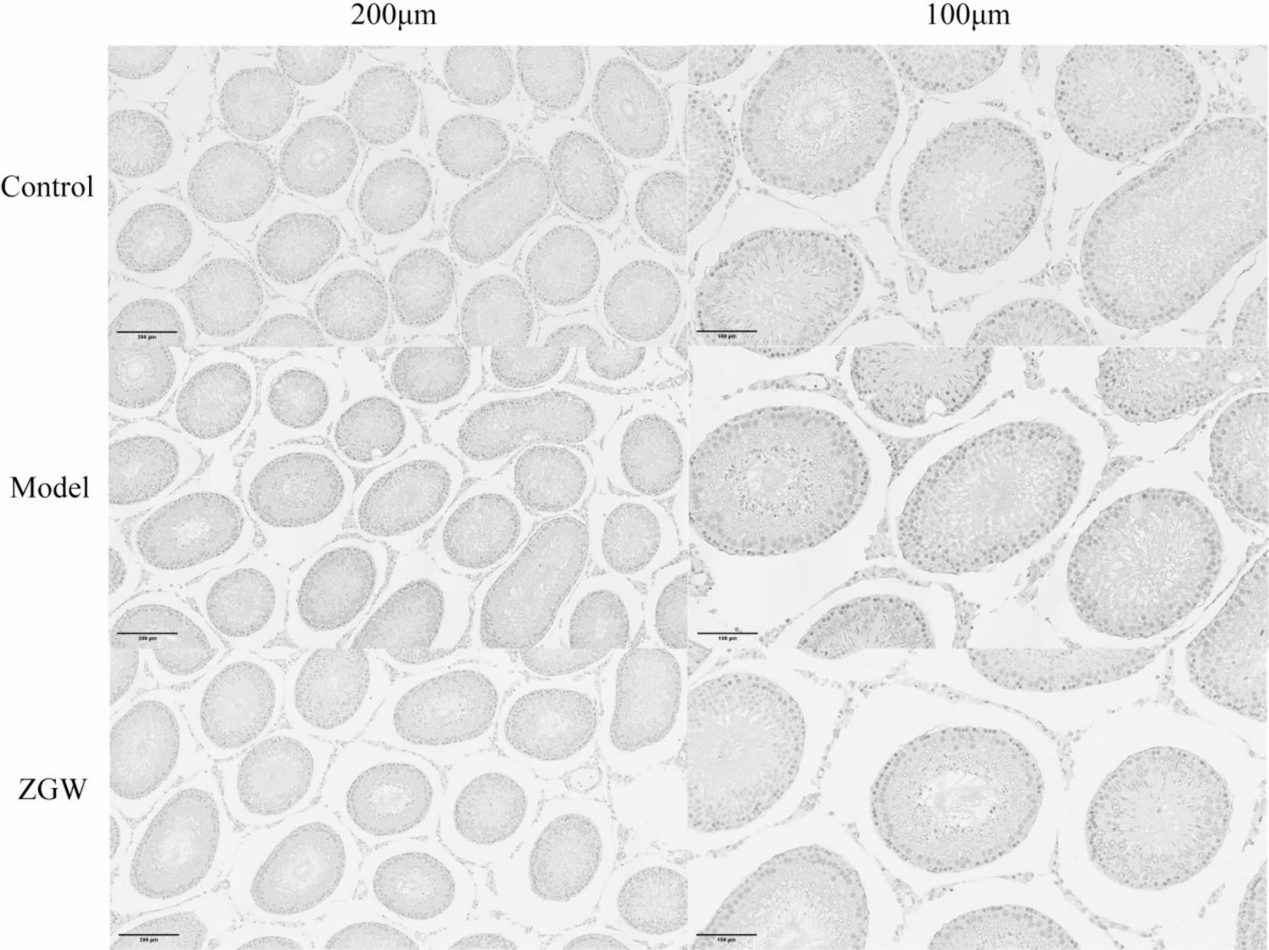
TUNEL staining showed the protective effect of ZGW on apoptosis in the testicular tissue of rats.

TUNEL staining results of rat testicular tissue under different treatment conditions, used to assess cell apoptosis, are shown in Fig. 11. To facilitate comparison, each group is presented at magnifications of 200 μm and 100 μm. The results indicate that the control group exhibited few apoptotic signals, the model group showed a significant increase in apoptosis, while the ZGW group displayed a marked reduction in apoptosis compared to the model group.

In the model group, the seminiferous tubules displayed severe structural abnormalities, including disrupted arrangement, reduced lumen size, epithelial degeneration, and numerous vacuoles. Mature sperm were scarce, and the proportion of abnormal sperm and spermatogenic cells was markedly increased, with extensive spermatogenic cell loss and disorganized structures observed. In contrast, the ZGW group showed significantly reduced severity and extent of pathological changes. The seminiferous tubule lumen was visibly expanded, sperm count increased noticeably, and some seminiferous tubules regained a more normal structure, although certain differences remained compared to the control group. These findings indicate that the model group suffered significant testicular cell apoptosis and structural damage, while ZGW treatment provided protective effects by reducing apoptosis and preserving testicular cell integrity, supporting its potential therapeutic effect on OAS.

### WB and Q-PCR results in *vivo*

**Fig. 12:**
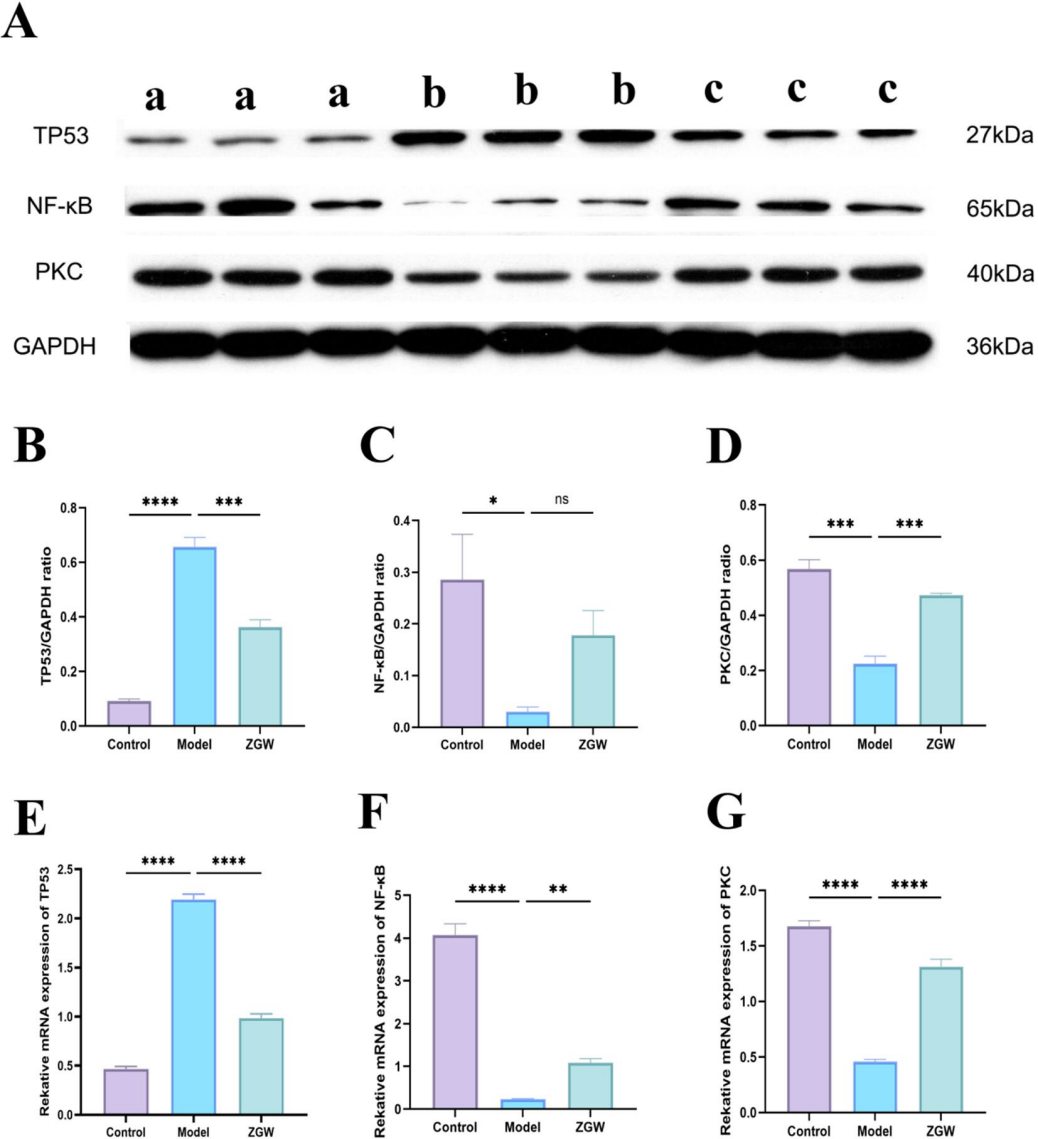
WB and Q-PCR results of in *vivo* experiments.(**A**) WB analysis of P53, NF-kB, and PKC proteins in the ZGW-OAS model.a: control group, b : model group, and c : Zuo Gui Wan group.(**B**) Expression of TP53 protein in each group.(**C**) Expression of NF-kB protein in each group.(**D**) Expression of PKC protein in each group.(**E**) TP53 mRNA expression in each group.(**F**) NF-kB mRNA expression in each group.(**G**) PKC mRNA expression in each group.ns *P* > 0.05; * *P* ≤ 0.05; ** *P* ≤ 0.01; *** *P* ≤ 0.001; **** *P* ≤ 0.0001.

Building on the in *vitro* experiments, in *vivo* studies further confirmed the effects of ZGW. Western blot results (Figs. 12A-D) showed that, consistent with in *vitro* findings, TP53 expression was highest in the model group and significantly reduced after ZGW treatment. NF-κB and PKC expression were highest in the control group, followed by the ZGW group, and lowest in the model group. Q-PCR results (Figs. 11E-G) also confirmed this trend. TP53 expression was slightly higher in the ZGW group compared to the control group but lower than in the model group, while NF-κB and PKC expression were slightly lower in the ZGW group than in the control group and significantly higher than in the model group. The in *vivo* results corroborated the in *vitro* findings, further confirming that ZGW modulates the expression of TP53, NF-κB, and PKC, suggesting that it may improve the pathological state by influencing these proteins.

## Discussion

With the rapid pace of modern life, changes in lifestyle, and increasing environmental pollution, male infertility, especially OAS, has become a global public health issue. The complex pathogenesis of OAS involves hormonal dysregulation, increased oxidative stress, apoptosis, and autophagy dysfunction, all of which are key targets in modern pharmacological research. However, traditional single-target drug therapies often have limitations. In contrast, TCM adopts a multi-pharmacological, multi-target, and multi-pathway approach. This method not only effectively alleviates the apoptosis of testicular germ cells and regulates endocrine function but also restores the normal process of spermatogenesis. More importantly, through holistic regulation and individualized treatment, TCM significantly promotes semen liquefaction and enhances sperm motility, demonstrating its unique therapeutic advantages. This study combines network pharmacology and molecular docking methods to predict the key components and target proteins of ZGW in the treatment of OAS. In vivo and in vitro experiments further validate the potential therapeutic mechanisms of ZGW in a rat model of OAS, providing scientific evidence for its further development and application.

At present, the etiology and pathogenesis of OAS remain unclear. The 182 common targets identified through Venny software demonstrate strong relevance to OAS. PPI network analysis and GO enrichment analysis indicate that these targets are primarily involved in hormonal responses, protein kinase activity, and cell membrane structure. This suggests that the therapeutic effects of ZGW may regulate spermatogenesis, sperm survival, and sperm motility through cellular stress responses, hormonal regulation, and signal transduction pathways. Research has shown that sperm production is closely associated with oxidative stress, apoptosis, and autophagy. As a key regulatory mechanism in spermatogenesis and maturation, autophagy plays a role in both biosynthesis and catabolism. However, whether autophagy serves as a protective or detrimental mechanism in these processes requires further exploration^46^. Additionally, apoptosis, an essential process for maintaining biological homeostasis and normal development, plays a crucial role in various diseases and their treatment^47^.In addition, sperm motility is primarily driven by energy from mitochondria, which not only serve as the main sites for oxidative metabolism and free radical production but also participate in critical apoptotic processes^48^. An abnormal increase in reactive oxygen species (ROS) can lead to changes in mitochondrial membrane potential, sperm nuclear DNA fragmentation, and lipid peroxidation of the plasma membrane, ultimately impairing sperm function^49^. In this study, we further validated the therapeutic effects of ZGW through ultrastructural observations and histopathological analysis in a rat model of OAS. The results showed that ZGW significantly mitigated cellular damage in testicular tissue, reduced apoptosis, and restored the normal structure and function of seminiferous tubules. These findings suggest that ZGW may treat OAS by inhibiting ROS, alleviating oxidative stress, and regulating apoptosis and autophagy.

The expression of hormones is closely associated with the secretion and maturation of germ cells. It may optimize the microenvironment of spermatogenesis by regulating apoptosis and maintaining communication between germ cells, thus further influencing sperm production and quality. Research has shown that oxidative stress can affect male gonadal function through multiple pathways, particularly by modulating hormonal regulation, which exacerbates spermatogenic disorders and reproductive dysfunction^50^. Furthermore, excessive ROS induced by oxidative stress can impair testosterone synthesis, trigger spermatogenic cell apoptosis, decrease sperm concentration and motility, and damage sperm morphology and DNA integrity^51^. Sperm quantity and quality depend on various stages of spermatogenesis, particularly the differentiation of spermatogonia into mature sperm. In this process, sperm differentiation and development are hormonally regulated, with hormones such as adrenaline, luteinizing hormone (LH), and follicle-stimulating hormone (FSH) playing vital roles in regulating testicular function and spermatogenesis. Any factors that interfere with this process, such as renal dysfunction or endocrine disorders, can result in reduced sperm count and semen quality^52^. Androgens and estrogens play crucial roles in the regulation of male reproductive function^53^. Androgens not only have a broad impact on muscle physiology, lean body mass regulation, fat metabolism, bone density maintenance, and cognitive function but also occupy a central role in spermatogenesis, male reproductive health, and the maintenance of sexual function^54^. Similarly, estrogens are indispensable in the development and regulation of male reproductive function, acting primarily through ligand-dependent transcription factors, such as estrogen receptor 1^55^. Studies have shown that male mice lacking estrogen receptor 1 exhibit significant sperm abnormalities and reduced fertilization rates. The primary mechanism is that the reabsorption of fluid in the seminiferous tubules is inhibited, leading to fluid accumulation in the lumen, increased backpressure, and eventually testicular degeneration and severe spermatogenic disorders^56^. TCM theory emphasizes that sperm generation is closely linked to “kidney essence,” and the kidney is considered the “foundation of congenital vitality” and the “master of reproduction.” The fullness of kidney essence directly impacts reproductive function. The occurrence of oligoasthenozoospermia is often associated with “kidney essence deficiency” and “congenital insufficiency.” Numerous studies have shown that ZGW can regulate hormone expression levels. Our results confirm that ZGW improves the cellular structure and function of oligoasthenozoospermia model rats, and its mechanism may be linked to the regulation of hormone levels. Further exploration is needed to investigate how ZGW, through its “nourishing yin and replenishing the kidney, filling essence and improving marrow” actions, regulates hormone expression and consequently modulates testicular function and spermatogenesis to treat oligoasthenozoospermia.

KEGG analysis suggests that ZGW may promote spermatogenesis and semen liquefaction by modulating multiple classical signaling pathways. Among these, the regulation of the PI3K/AKT and MAPK signaling pathways is crucial in male reproductive health. Specifically, the PI3K/AKT signaling pathway plays a key role at various stages of male reproduction, including regulating the hypothalamic-pituitary-gonadal axis, promoting the proliferation and differentiation of testicular cells, protecting testicular cells from apoptosis, supporting the survival of germ cells, and regulating the proliferation and differentiation of spermatogonial stem cells and supporting cells. It also modulates sperm autophagy in the presence of environmental pollutants, thereby influencing sperm production and maturation^57^. Studies have shown that local growth factors such as insulin-like growth factor-I, insulin-like growth factor-3, retinoic acid, and prohibitin can regulate this pathway to influence the behavior of spermatogonia, which is essential for maintaining the proper quantity and quality of spermatogonia in the testes^58–64^. Furthermore, under pathological conditions, the phosphorylation levels of the PI3K/AKT pathway are significantly reduced in infertile males, leading to impaired sperm mitochondrial function and decreased sperm motility^65^. The MAPK signaling pathway is involved in sperm development by controlling the signal transduction of DNA synthesis, cell division, and differentiation, regulating testicular transcription, cytoplasmic specialization, and the flagellar motility and acrosomal reaction of mature sperm^66–69^. It influences sperm function and structural integrity through the regulation of intracellular phosphorylation, cell cycle progression, and apoptosis^70^, while also repairing the blood-testis barrier^41^, ultimately impacting sperm fertilization ability.

To further investigate the key mechanisms underlying the treatment of OAS with ZGW, we constructed a drug-target network and identified 10 core components, among which quercetin, kaempferol, betavulgarin, and cyasterone exhibited the highest degree values. Literature review suggests that these core components enhance semen liquefaction, promote sperm differentiation, and improve motility, either directly or indirectly, by providing essential energy for sperm and improving fertility. Furthermore, molecular docking confirmed that these components form stable binding complexes with core proteins, reinforcing their importance. Among them, quercetin and kaempferol, two potent flavonoids, exhibit diverse biological activities such as antioxidant, anti-inflammatory, immunomodulatory, and anti-apoptotic effects, demonstrating significant application value in the treatment of male reproductive disorders^71,72^. Specifically, quercetin protects mitochondrial structure, ROS leakage, and decreases genomic susceptibility to oxidative damage, thereby improving sperm survival and spermatogenesis^73,74^. Notably, studies have shown that quercetin alleviates testicular oxidative stress, reduces apoptosis, and regulates the hypothalamic-pituitary-gonadal axis, contributing to enhanced sperm motility and dynamics^75–78^. Similarly, kaempferol exerts protective effects on male reproductive health by eliminating free radicals, regulating amino acid and lipid metabolism, and promoting spermatogenesis through key metabolic pathways^79,80^. Additionally, betavulgarin and cyasterone, derived from beetroot and Chinese foxglove, respectively, exhibit significant antioxidant and cell-protective effects. Research has demonstrated that betavulgarin protects mitochondrial structure, reduces oxidative stress, and improves sperm survival and quality^81^, while cyasterone, known for its anti-inflammatory and anti-tumor properties, plays a crucial role in regulating NF-κB and MAPK signaling pathways^82^. Moreover, cyasterone has been shown to upregulate the Nrf2 pathway through AKT/GSK3βphosphorylation, thereby inhibiting inflammation and oxidative stress and enhancing cellular stress resistance^83^. Structural visualization with PyMOL further confirmed the formation of stable hydrogen bonds, electrostatic forces, and hydrophobic interactions between cyasterone and P53 (8SVG), NF-κB (8TQD), and PKC (3IW4). Although molecular dynamics simulations indicate that cyasterone stably binds to P53 and NF-κB, its binding with PKC appears less stable. Nonetheless, hydrogen bond analysis shows sustained stability in all three complexes throughout the dynamic binding process. Although research on cyasterone’s role in OAS is still in its early stages, the aforementioned findings highlight its potential therapeutic effects via modulation of the PI3K-AKT, MAPK, and NF-κB signaling pathways. Additionally, cyasterone exhibits broad biological applications, including its role as a natural EGFR inhibitor in suppressing cancer cell growth^84^ and its ability to promote the growth of diamondback moth larvae at low concentrations^85^. Collectively, these findings provide strong evidence supporting cyasterone’s potential in treating OAS. However, further studies are needed to elucidate its precise mechanisms and clinical applications, particularly in maintaining sperm quality and overall reproductive health.

PKC, NF-κB, and P53 proteins are closely linked to the MAPK and PI3K-AKT signaling pathways, forming a highly interwoven regulatory network through phosphorylation modification, transcriptional regulation, protein-protein interactions, and feedback loops. These mechanisms play crucial roles in sperm production and physiological responses. Among them, P53 regulates the sperm life cycle, particularly in apoptosis^86^. During spermatogenesis, moderate P53 expression eliminates damaged or mutated spermatogonia, maintaining germ cell health and ensuring sperm quality^87–90^. However, excessive P53 activation leads to excessive apoptosis, negatively affecting sperm quantity and quality. Studies have shown that activation of the PI3K/AKT pathway downregulates P53 stability and activity, inhibiting its pro-apoptotic function and protecting spermatogenic cells from excessive apoptosis. In contrast, the JNK and p38 branches of the MAPK pathway phosphorylate P53, enhancing its transcriptional activity and promoting apoptosis under stress conditions^91,92^. Similarly, NF-κB, as a key transcription factor, regulates inflammatory responses and cell survival. The PI3K/AKT pathway activates NF-κB through the IKK complex, inducing anti-apoptotic gene expression, protecting spermatogonia, and supporting normal sperm development^45,93^. Additionally, PKC, primarily activated by diacylglycerol and intracellular Ca2 + release via IP3^94^, phosphorylates multiple kinases, including MAPK, PI3K-AKT, and NF-κB, thereby mitigating inflammation, oxidative stress, and apoptosis^95,96^.

In this study, we confirmed through in vitro and vivo experiments using WB and qPCR that ZGW significantly regulates the expression of key proteins such as TP53, NF-κB, and PKC, providing molecular evidence for its role in treating OAS. Our results showed that TP53 expression was highest in the model group, indicating its crucial role in promoting spermatogenic cell apoptosis during sperm production disorders. Excessive apoptosis disrupts the dynamic balance of spermatogenesis, reducing sperm quantity and quality. However, in the ZGW intervention group, TP53 expression significantly decreased, suggesting that ZGW reduces unnecessary spermatogenic cell apoptosis by inhibiting P53-mediated apoptotic pathways, thus improving testicular spermatogenic function. Conversely, NF-κB and PKC expression levels were lowest in the model group, indicating their inhibition might be associated with stagnation of spermatogenesis and impaired reproductive function. Notably, the ZGW intervention group exhibited significantly increased expression of NF-κB and PKC, suggesting that ZGW activates these proteins to promote spermatogenic cell survival, proliferation, and differentiation, ultimately enhancing sperm quality and motility. Combined with previous literature, these findings further highlight the pivotal role of P53 in cell apoptosis, cell cycle regulation, and DNA repair, while NF-κB and PKC pathways are crucial in testicular heat stress-induced germ cell apoptosis, inflammation, and acrosome reactions. Both in vitro and in vivo experiments demonstrate that ZGW not only alleviates P53-mediated excessive apoptosis but also improves oxidative stress and inflammation by activating NF-κB and PKC, thereby restoring normal sperm production. These results strongly suggest that ZGW exerts therapeutic effects on OAS by modulating PI3K-AKT and MAPK signaling pathways.

## Conclusion

In conclusion, our study demonstrates that ZGW significantly improves testicular tissue damage in rats with OAS through its multi-target and multi-pathway regulatory effects. It reduces cell apoptosis and restores the normal structure and function of seminiferous tubules, thereby enhancing male fertility. The underlying mechanisms likely involve the regulation of oxidative stress, apoptosis, autophagy, hormone regulation, and key signaling pathways such as PI3K-AKT and MAPK. Additionally, the expression of key proteins like TP53, NF-κB, and PKC is significantly modulated. The study also highlights the key components of ZGW—Quercetin, Kaempferol, Betavulgarin, and Cyasterone—with Cyasterone, in particular, standing out due to its strong interaction with protein targets and excellent molecular dynamics results. WB and PCR experiments suggest that ZGW exerts its therapeutic effects by inhibiting TP53 and activating the NF-κB and PKC signaling pathways. Although ZGW shows significant therapeutic efficacy, further research is needed to fully elucidate its specific roles in different signaling pathways and hormonal regulation, as well as to optimize treatment strategies, particularly focusing on its regulation of sperm production. This study provides a new candidate for the treatment of OAS and offers novel insights into the use of traditional Chinese medicine in treating male infertility.

## Supplementary Information

Below is the link to the electronic supplementary material.

Supplementary Material 1

Supplementary Material 2

Supplementary Material 3

## Data Availability

No datasets were generated or analysed during the current study.

## Abbreviations

OAS: Oligoasthenozoospermia
ZGW: Zuogui Wan
TCMSP: Traditional Chinese Medicine Systems Pharmacology database and analysis platform
OMIM: Online Mendelian Inheritance in Man
PPI: Protein-protein interaction
TCM: Traditional Chinese Medicine
GO: Gene Ontology
KEGG: Kyoto Encyclopedia of Genes and Genomes
MD: Molecular dynamics
PME: Particle mesh Ewald
RMSD: Root Mean Square Deviation
Rg: Radius of Gyration
SASA: Solvent Accessible Surface Area
RMSF: Root Mean Square Fluctuation
TEM: Transmission electron microscopy
HE: Hematoxylin & eosin
GTW: Tripterygium wilfordii multiglycoside
WB: Western Blot
RT-qPCR: Real-Time Quantitative PCR
ROS: Reactive oxygen species
