# Supplementary figures and images for: The therapeutic potential of Zuogui Wan in oligoasthenozoospermia: insights from network pharmacology, molecular docking, molecular dynamics simulation, and experimental validation
Source: Sci Rep. 2025 Nov 4;15:38576. doi: 10.1038/s41598-025-22348-w (PMC12586514; doi:10.1038/s41598-025-22348-w)

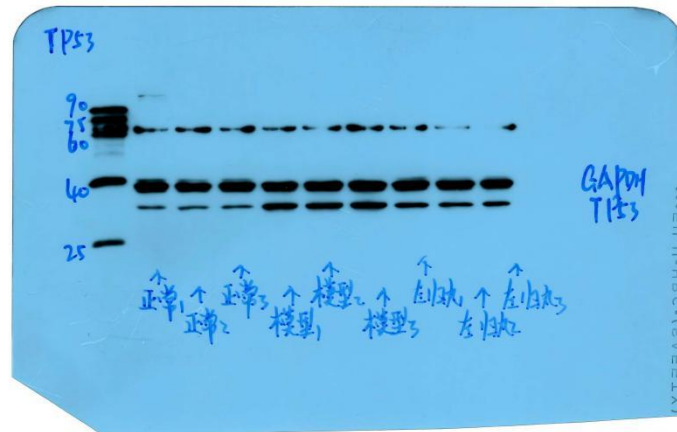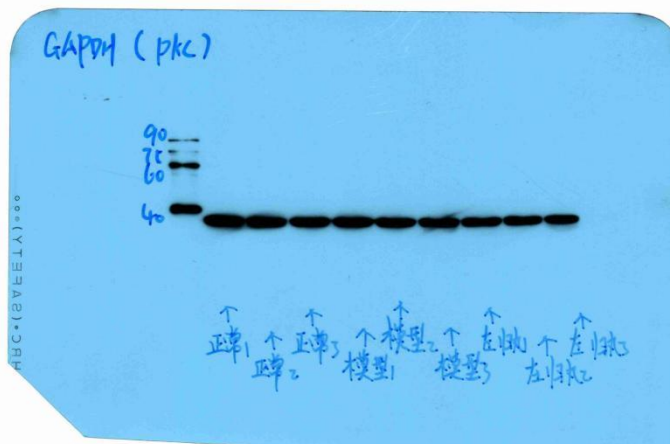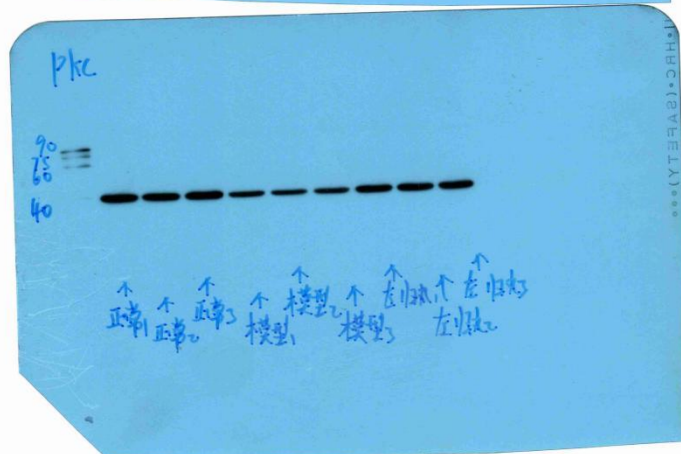

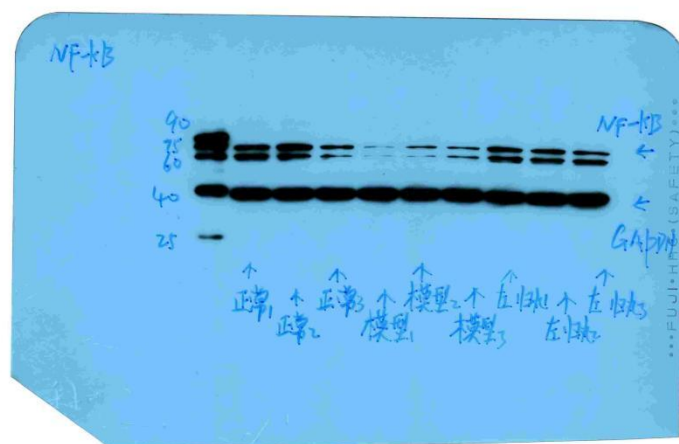

Supplement: Supplementary file 1 — Supplementary Material 1 [file 41598_2025_22348_MOESM1_ESM.pdf]

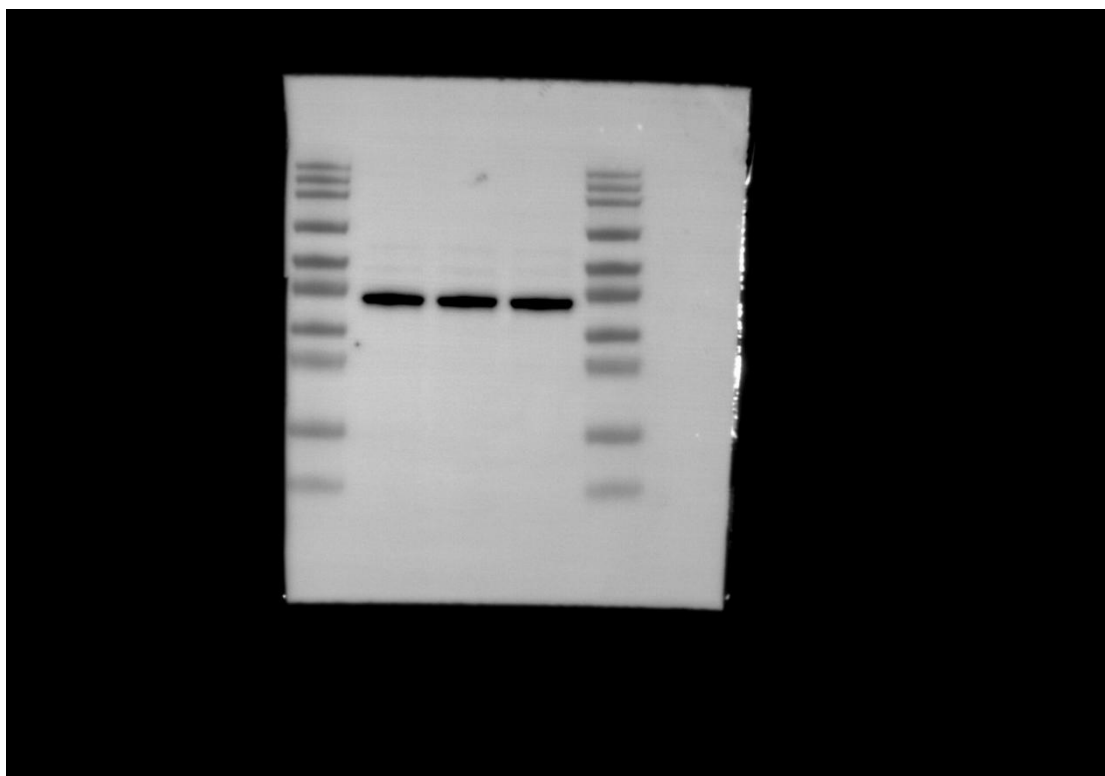

GAPDH

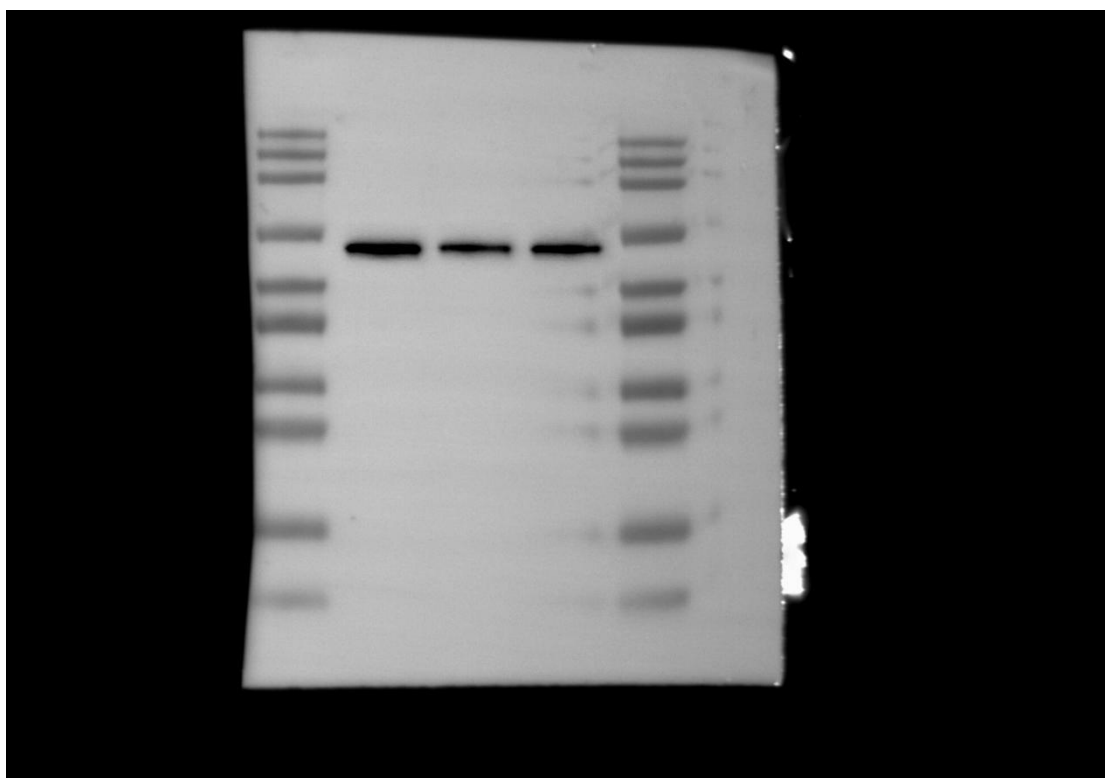

P65 (NFkB)

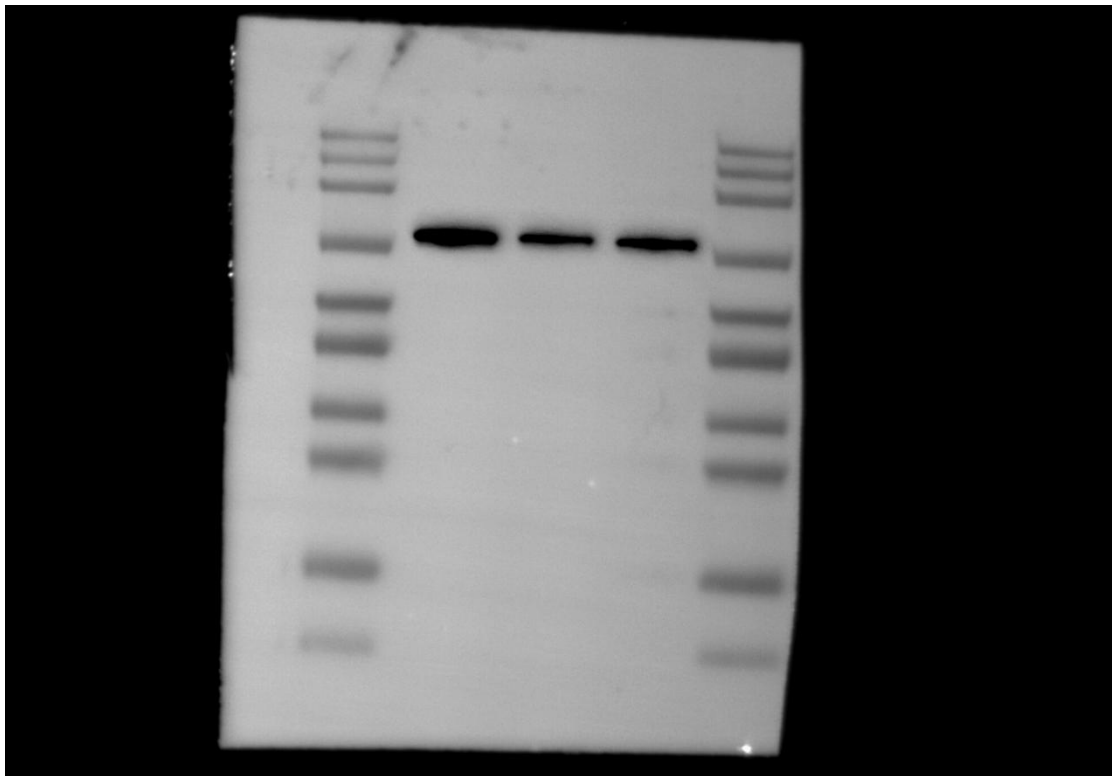

PKC

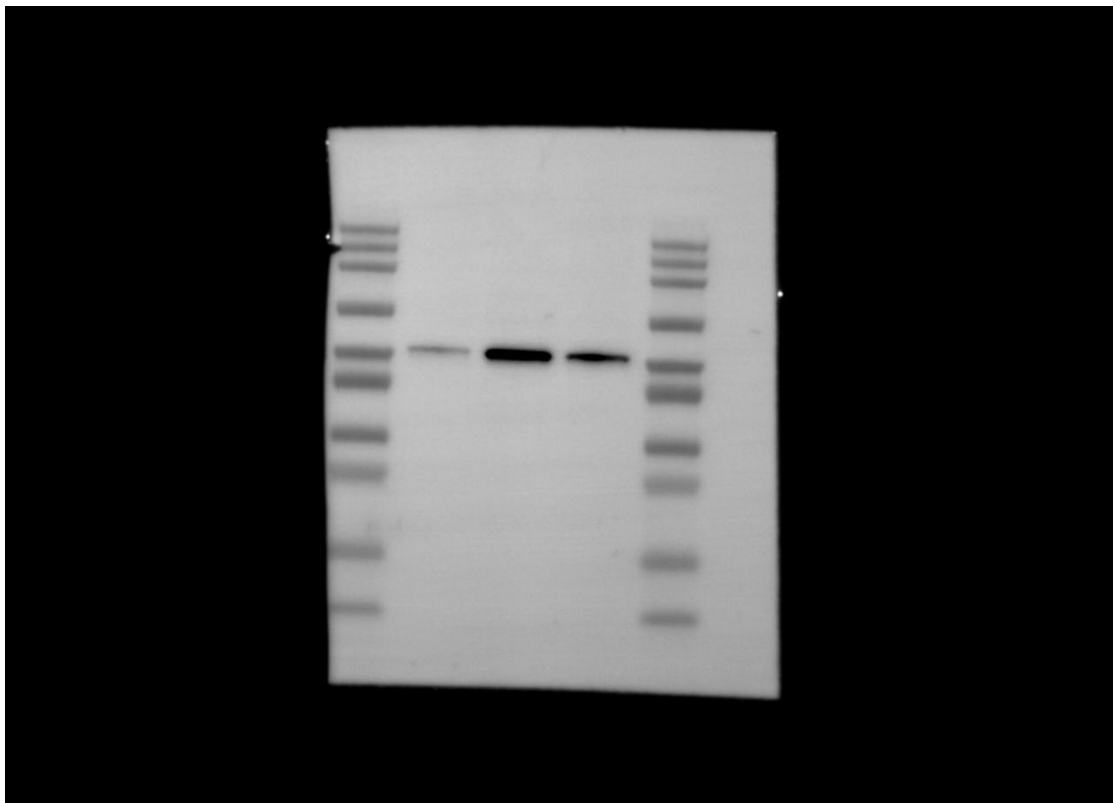

TP53

Supplement: Supplementary file 2 — Supplementary Material 2 [file 41598_2025_22348_MOESM2_ESM.pdf]
