# Supplementary material for: The therapeutic potential of Zuogui Wan in oligoasthenozoospermia: insights from network pharmacology, molecular docking, molecular dynamics simulation, and experimental validation
Source: Sci Rep. 2025 Nov 4;15:38576. doi: 10.1038/s41598-025-22348-w (PMC12586514; doi:10.1038/s41598-025-22348-w)
Supplement: Supplementary file 3 — Supplementary Material 3 [file 41598_2025_22348_MOESM3_ESM.docx]

Supplementary Material

## Supplementary Figures


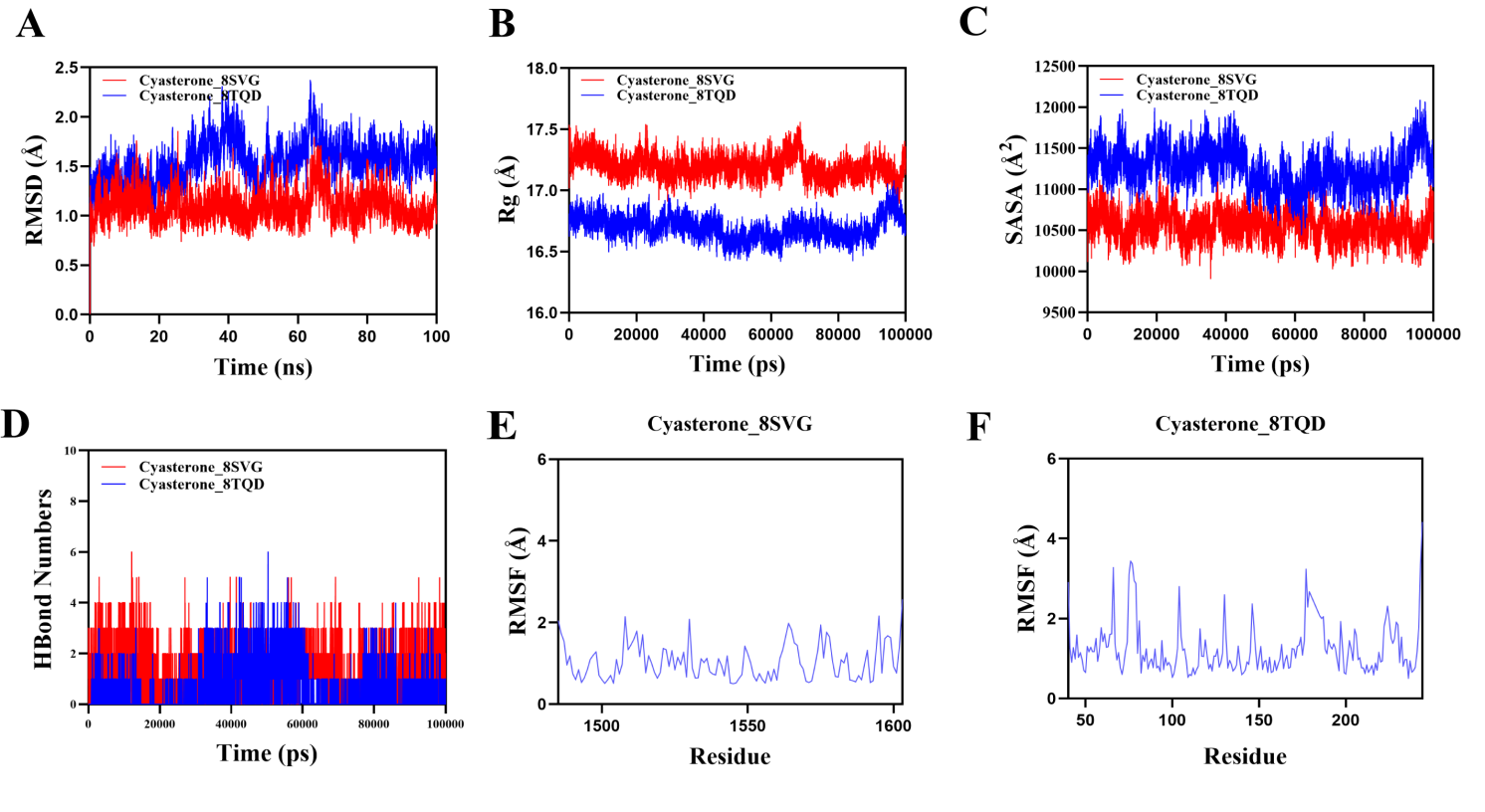


**Supplementary Figure 1.** Molecular dynamics simulation of key compounds and core target proteins.(A) Root Mean Square Deviation (RMSD) analysis of Cyasterone_8SVG and Cyasterone_8TQD.(B) Radius of Gyration (Rg) analysis of Cyasterone_8SVG and Cyasterone_8TQD.(C) Solvent Accessible Surface Area (SASA) analysis of Cyasterone_8SVG and Cyasterone_8TQD.(D) Hydrogen Bond Numbers (HBond Numbers) analysis of Cyasterone_8SVG and Cyasterone_8TQD.(E) Root Mean Square Fluctuation (RMSF) analysis of Cyasterone_8SVG.(F) Root Mean Square Fluctuation (RMSF) analysis of Cyasterone_8TQD.


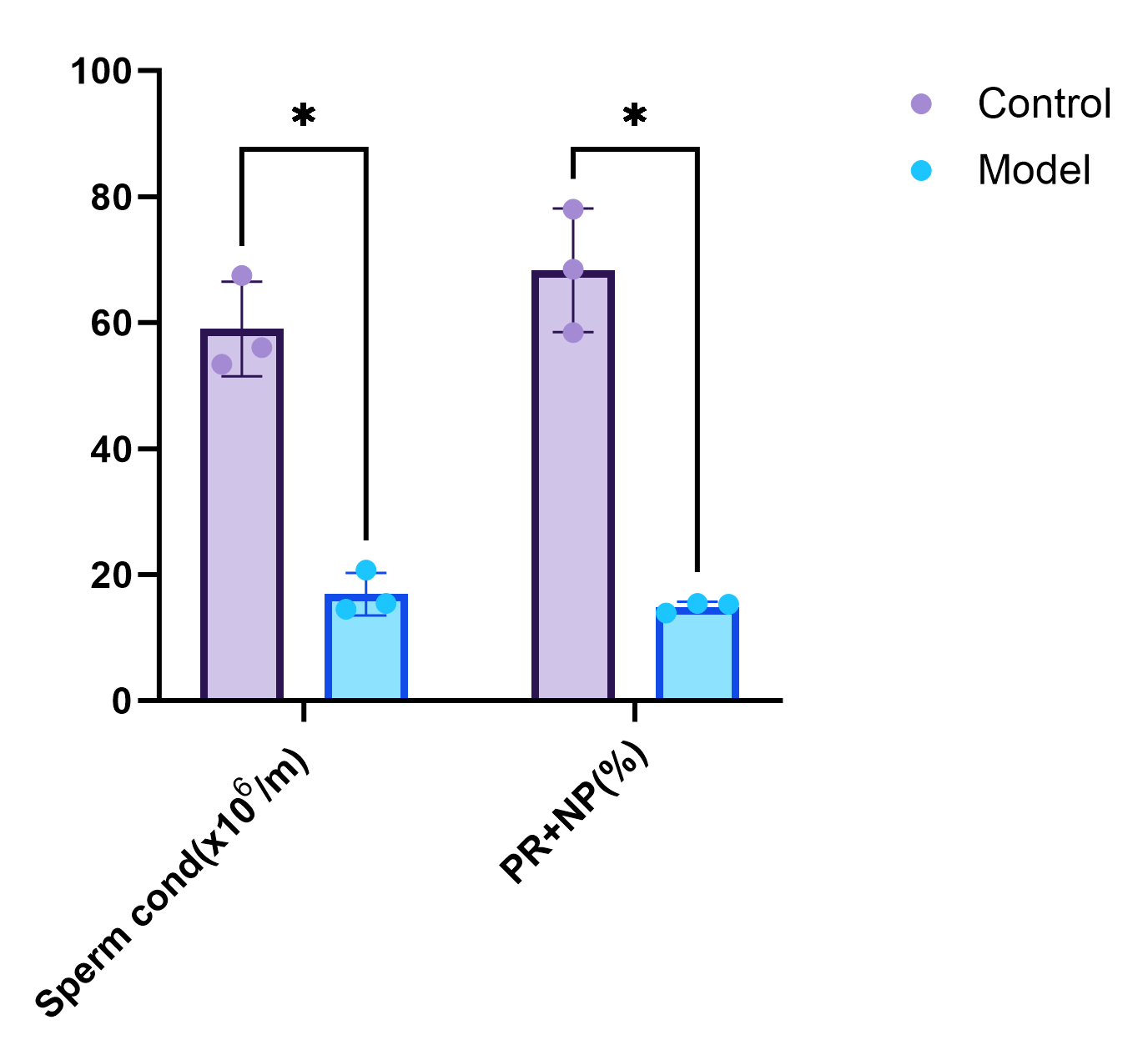


**Supplementary Figure 2.**Comparison of sperm concentration and total motility between the blank group and the model group.
